# Long‐Term Real‐World Utilization and Continuity Patterns in Injectable Aesthetic Dermatology: A 10‐Year Single‐Center Study

**DOI:** 10.1111/jocd.71234

**Published:** 2026-10-14

**Authors:** Ronghao Ouyang, Ximeng Jia, Ziming Zhang, Yutong Liang, Benjie Li, Mengzhe Qin, Jintian Hu

**Affiliations:** ^1^ Department of Cosmetic Injection Center Plastic Surgery Hospital, Chinese Academy of Medical Sciences and Peking Union Medical College Beijing China; ^2^ Laser Aesthetic Center Plastic Surgery Hospital, Chinese Academy of Medical Sciences and Peking Union Medical College Beijing China

**Keywords:** continuity of care, injectable aesthetic dermatology, maintenance utilization, outpatient care, patient geographic origin, patient retention, real‐world evidence

## Abstract

**Background:**

Injectable aesthetic dermatology increasingly depends on repeated maintenance care, yet long‐term real‐world evidence on utilization and continuity is limited.

**Aims:**

To characterize long‐term real‐world utilization in injectable aesthetic dermatology, with attention to maintenance cadence, patient‐origin composition, and continuity of care.

**Methods:**

We retrospectively analyzed outpatient registration and billing records from one center (2015–2024). Geographic origin was an administrative address category, not measured travel burden. Annual visit‐frequency comparisons used patient‐years from 2022 to 2024; retention used index years 2015–2023, with a recent‐window analysis. Patient‐year models used standard errors clustered by patient. Time to second visit was analyzed by Kaplan–Meier and Cox methods, overall and by strictly recorded first‐visit indication proxy. Billing outcomes were exploratory.

**Results:**

Among 48 798 visits in 29 437 patients, service volume increased, especially in 2023–2024. The proportion with ≥ 2 visits/year rose from 17.6% to 27.2%, with a positive trend after age and sex adjustment. Most patient‐years in each origin group comprised one visit (75.5%–77.2%); adjusted annual count differences were small. Among 25 399 patients with resolvable origin, 29.8% had a second observed visit. Distant nonlocal origin was associated with a lower second‐visit hazard (adjusted HR 0.83, 95% CI 0.78–0.88); proportional hazards were imperfect, so time‐specific estimates are also reported. The origin‐by‐indication interaction was exploratory (*p* < 0.001). Recent‐window next‐year retention was 26.0%, 21.9%, and 21.2% in Beijing, nearby nonlocal, and distant nonlocal patient‐years.

**Conclusion:**

Repeat attendance increased, although single‐visit patient‐years remained common. Geographic origin had modest associations with observed re‐engagement at this center. These descriptive findings may inform prospective evaluation of follow‐up and communication strategies.

**Abbreviations**
aHR: Adjusted hazard ratio
CI: Confidence interval
COVID‐19: Coronavirus disease 2019
HR: Hazard ratio
IQR: Interquartile range
OR: Odds ratio
RMB: Renminbi (Chinese yuan)
RR: Rate ratio
STROBE: Strengthening the Reporting of Observational Studies in Epidemiology

## 1 Introduction

Injectable aesthetic dermatology has become a longitudinal, maintenance‐based model of care. It requires repeated visits for treatment planning, effect maintenance, and patient counseling. Professional society reports continue to identify minimally invasive procedures as a cornerstone of contemporary aesthetic practice [1, 2]. The effects of neuromodulators and dermal fillers are time‐limited, which gives a clinical basis for repeated visits and a benchmark against which real‐world attendance can be compared [3, 4, 5, 6, 7, 8, 9]. Unlike one‐time procedural encounters, maintenance‐based injectable care depends on repeated patient–clinic contact. It therefore raises practical questions about utilization cadence, continuity, and service organization.

Patient residential origin may serve as a pragmatic descriptor of a service's catchment composition. Without travel‐time or contextual data, geographic origin should not be read as a direct measure of travel burden or accessibility. It may still help practices understand how repeated aesthetic care is organized among local and nonlocal patients [10, 11, 12, 13, 14].

Time‐varying influences during and after the COVID‐19 pandemic are also relevant to elective aesthetic dermatology. Google Trends studies show large shifts in public interest in plastic and aesthetic procedures across phases of restriction and reopening [15, 16]. Survey evidence has linked greater video‐calling exposure to wider acceptance of aesthetic surgery, the so‐called “Zoom effect” [17]. In China, the medical aesthetics market has expanded rapidly [18], within a formally defined regulatory framework for aesthetic injection devices [19], and amid growing scholarly attention to the management and legal dimensions of aesthetic medicine [20]. Chinese studies have also begun to characterize practitioner training needs, patient risk awareness, and region‐specific injection strategies [21, 22, 23].

Prior work has examined product‐specific efficacy, duration, retreatment, real‐world product use, multimodal treatment, and patient‐reported satisfaction after botulinum toxin or hyaluronic acid filler treatment [3, 4, 5, 6, 24, 25, 26, 27]. Much less is known about long‐term, practice‐level utilization patterns and continuity indicators. Using a decade of real‐world outpatient registration and billing data, this study therefore aimed to characterize temporal trends in injectable aesthetic dermatology utilization, and to examine whether patient geographic origin was associated with maintenance cadence and continuity of care.

## 2 Methods

### 2.1 Study Design and Setting

This was a single‐center, retrospective observational analysis. It used routinely generated outpatient registration and billing data from the injectable aesthetic dermatology service of a tertiary academic plastic surgery center in Beijing, China. The study period ran from January 1, 2015 to December 31, 2024. Reporting follows the Strengthening the Reporting of Observational Studies in Epidemiology (STROBE) statement [28], and a completed STROBE checklist is provided as Table S5 in Appendix S1.

The center is a public, hospital‐based specialty service. According to the service description, most patients attend on their own initiative and no structured inter‐institutional referral pathway operates for aesthetic injectables. Informal referrals, professional recommendations, and online reputation were not recorded and cannot be excluded as influences on attendance.

Throughout this report, “the COVID‐19 pandemic period” refers to calendar years 2020–2022, and “the post‐pandemic period” refers to 2023–2024; the term “pandemic” is not used in any other sense.

### 2.2 Terminology

Because several terms describe measures constructed from administrative data rather than established clinical constructs, we define them explicitly.

– *Maintenance‐based model of care*: a pattern of care in which the effect of the index treatment is time‐limited, so that sustaining it requires planned repeat treatment rather than a single definitive procedure. This is a property of the modality, not a claim about any patient's treatment plan.
– *Cadence*: the number of recorded visits within one calendar year (annual visit count and categories 1, 2, ≥ 3 visits/year), among patient‐years with at least one encounter. It does not condition on a second visit or establish a planned maintenance schedule.
– *Continuity of care*: used only in the operational sense of observed re‐engagement with this service, measured by three indicators: returning patient‐year (attendance in any earlier calendar year), year‐over‐year retention (attendance in the following calendar year), and time to second observed visit. It does not denote relational or informational continuity as used in primary‐care research, and cannot capture care delivered elsewhere.
– *Patient‐origin composition*: the distribution of the service's patients across origin groups in a given period, that is, catchment composition as recorded administratively. It is a property of the service population, not of a patient.
– *Indication proxy*: a category inferred from the recorded primary diagnosis, approximating the class of modality clinically indicated; not a record of what was administered.
– *Treatment visit*: an encounter with a recorded charge above the chosen billing threshold. This is an operational billing proxy, not confirmation of an administered procedure or a measure of treatment intensity. Missing charges remain unclassified.

### 2.3 Data Source and Data Extraction

Data were extracted as a single query from the hospital's outpatient information system. The query covered all encounters recorded under the injectable aesthetic dermatology service between January 1, 2015 and December 31, 2024, and returned one row per encounter with nine structured fields: a de‐identified unique patient identifier, an encounter serial number, a de‐identified medical record number, sex, date of birth, encounter date and time, the free‐text primary outpatient diagnosis, the structured residential address recorded at registration (province/city/district), and the total visit‐level charge. Field completeness across the 48 798 extracted encounters is reported in Table S9.

The available extract contained no product‐level treatment mix, procedure codes, dose, package‐level billing, physician identifiers, clinical outcomes, patient satisfaction, or adverse events. No linkage to other hospital systems was performed for the present analysis.

Derivation was performed in Python. Annual visit and unique‐patient counts were reconciled with the submitted aggregate summaries, and the fixed province‐to‐region and diagnosis mappings were retained. For the correction check, patient‐year measures and first‐to‐second‐visit outcomes were rebuilt from the available encounter‐level snapshot. Appendices S2 and S3 provide post‐extraction processing and analysis code, including explicit censoring, missing‐data handling, and model definitions. The code does not reproduce the hospital database query. These checks do not constitute independent validation against the original hospital database or a treatment register.

To characterize maintenance utilization and exploratory billing‐based patterns, we constructed a patient‐year dataset by consolidating all encounters of the same patient within each calendar year into a single record. For each patient‐year, we derived: (i) annual visit count; (ii) maintenance categories (1 visit, 2 visits, ≥ 3 visits), along with the proportions of ≥ 2 visits/year and ≥ 3 visits/year; (iii) annual total cost (RMB), defined as the sum of visit‐level charges; and (iv) cost per visit, calculated as annual total cost divided by annual visit count. Patient‐years with any missing encounter charge or annual total cost of zero were excluded from cost‐related descriptive analyses and cost models, but were retained for visit‐frequency analyses. Charges were not imputed; incomplete annual sums were excluded from cost analyses.

### 2.4 Diagnosis‐Derived Indication Proxy Categories

Product‐level treatment mix was unavailable. We therefore derived indication proxy categories from the free‐text primary outpatient diagnosis recorded at each visit. This field contained 79 distinct diagnostic terms across the study period. Two clinicians independently mapped each distinct term to one of five prespecified categories, based on the anatomical problem described. Disagreements were resolved by discussion. The categories were:

(i) neuromodulator‐type indications, covering dynamic rhytides and muscle hypertrophy (glabellar lines, lateral canthal lines, forehead lines, masseter hypertrophy, gastrocnemius or posterior tibial muscle hypertrophy, platysmal banding, axillary hyperhidrosis); (ii) volume/contour‐type indications, covering soft‐tissue depressions and contour deficiencies (facial depression, nasolabial fold depression, tear‐trough or temporal hollowing, low nasal dorsum, thin lips, microgenia, facial asymmetry); (iii) skin‐quality‐type indications, covering photoaging and skin‐surface concerns (solar lentigines, cutaneous dryness, facial skin laxity); (iv) post‐procedure review, covering encounters coded as review after injection of facial implant material; and (v) other or unclassifiable.

These categories are indication proxies inferred from diagnostic coding. This is the single most important interpretive constraint on the present report. They were not derived from product names, procedure codes, or dose records. They were not validated against any treatment register, prescription record, or independent clinical review. They must not be read as measures of the products or procedures administered. Wherever an indication proxy appears below, it denotes the recorded anatomical problem, not the treatment given. As a face‐validity check only, we compared visit‐level charges across categories.

First, the primary diagnosis field was not recorded at every encounter. Availability was 54.1% in 2015, 81.2% in 2023, and 91.5% in 2024 (83.7% pooled across 2022–2024). Descriptive indication mix and annual cadence analyses use 2022–2024. Exploratory first‐visit survival strata use 2015–2024, and indication‐adjusted retention uses index years 2015–2023 with a recent‐window analysis. Patient‐year models adjust for calendar year categorically, whereas Cox models stratify baseline hazards by first‐visit calendar year. First‐visit diagnosis means the diagnosis on the first encounter row; a missing value is not replaced with a diagnosis from a later encounter.

Second, a single recorded diagnosis cannot represent a combination treatment. For patient‐years containing more than one category, a prespecified hierarchical rule assigned one category per patient‐year, in the order neuromodulator‐type > volume/contour‐type > skin‐quality‐type > post‐procedure review > other. Any ordering would be arbitrary, and this one systematically over‐attributes multi‐category patient‐years to earlier categories. We therefore quantified how often it operated. Among the 15 665 patient‐years with an assignable category in 2022–2024, 1574 (10.0%) contained more than one category (7.1% across the whole period). At most one patient‐year in 10 could therefore be reclassified under a different ordering. The distribution should still be read as *dominant recorded indication under a fixed rule*, not as the prevalence of treatment types. Indication‐stratified analyses are treated as exploratory throughout.

### 2.5 Treatment Visits Versus Consultation‐Only Visits

Encounters with a recorded charge were classified relative to the chosen threshold as billing‐positive (> 60 RMB in the primary analysis) or low‐charge (≤ 60 RMB). The operational terms treatment visit and consultation‐only visit refer to these billing proxies; encounters with missing charges were left unclassified.

The low‐charge distribution had an annual mode of 28 RMB (2015), 14 RMB (2016), 60 RMB (2017–2018), 50 RMB (2019–2023), and 60 RMB (2024) (Table S10). These are empirically identified low‐charge modes, compatible with registration/consultation fees; an audited fee schedule was unavailable. We retained 60 RMB as the primary fixed threshold and examined the year‐specific mode and fixed thresholds of 50, 100, and 200 RMB. No inflation adjustment was applied. Of all encounters, 33.8% had recorded charges ≤ 60 RMB; missing charges were not classified as consultation‐only. Consecutive billing‐positive encounter intervals describe attendance, not confirmed retreatment with the same product.

The threshold remains a billing proxy with two unavoidable limitations. It cannot distinguish an unbilled clinical review from a procedure billed under a separate package or prepaid course, and it does not measure treatment intensity. A procedure billed outside the encounter record is classified as consultation‐only, which inflates apparent retreatment intervals. A small ancillary charge at a review visit is classified as a treatment visit, which shortens them. The direction of net bias cannot be determined from these data.

### 2.6 Patient‐Origin Classification and Periodization

Residential origin was classified into patient‐origin groups for descriptive and comparative analyses. The primary regional classification for comparative analyses was a three‐level grouping intended to maximize interpretability for an international readership: Beijing (local), nearby nonlocal (Tianjin and Hebei), and distant nonlocal (all other non‐Beijing regions). A more granular six‐region classification was retained for descriptive characterization of catchment composition and for sensitivity analyses, and is reported in the Supporting Information: Beijing; Jing‐Jin‐Ji (Tianjin and Hebei); Other Eastern; Central; Western; and Northeast. Hong Kong SAR, Macau SAR, and Taiwan were mapped to Other Eastern. Provinces were assigned to macro‐regions according to a predefined province‐to‐region mapping used consistently across all analyses. Three‐stage periods were used to summarize patient‐origin composition across 2015–2019, 2020–2022, and 2023–2024.

Two properties of the address field materially constrain interpretation.

First, among the 25 414 patients with any recorded address, none had more than one distinct non‐missing address string in the available 10‐year extract (0.00%). No tie between different recorded addresses was encountered. A resolved province was propagated across the same patient's records when some encounters lacked a parsable address. This within‐patient reconciliation differs from imputing an unknown patient origin. The extract does not establish whether historical addresses were retained or overwritten in the source system; residence changes and secondary residences cannot be reconstructed.

Second, address information remained incomplete or unresolvable for 4038 of 29 437 patients (13.7%). These patients were excluded from origin comparisons but retained in non‐geographic summaries. Missingness was concentrated in early years: unresolvable origin affected 65.4% of patient‐years in 2015, 59.1% in 2016, 5.0% in 2019, and 0.40% across 2022–2024. Characteristics are compared in Table S11. The temporal pattern does not exclude other causes of missingness or selection bias.

Exact geocoded addresses, transportation mode, travel time, address‐change histories, and socioeconomic status were all unavailable. We therefore treat geographic origin as an administrative descriptor of patient‐origin composition, not as a measure of travel burden or spatial accessibility.

### 2.7 Outcomes

We defined outcomes at two analytic levels: (A) whole‐period descriptive outcomes (2015–2024), in which annual total visits and unique patients were summarized to characterize long‐term utilization trends and patient‐origin composition; and (B) patient‐year analytic outcomes (2022–2024), selected as the most recent practice phase for regional comparison and current service‐organization relevance.

Primary patient‐year outcomes were annual visit count, ≥ 2 visits/year, and ≥ 3 visits/year.

Continuity outcomes were defined as follows. A patient‐year was classified as a returning patient‐year if the same patient had appeared in the dataset in any previous calendar year, and as a first‐observed patient‐year otherwise. This definition is cumulative and does not require attendance in consecutive years: a patient who first appeared in 2015 and next appeared in 2024 is classified as a returning patient‐year in 2024, as is a patient who attended in 2023 and again in 2024. Because the dataset begins in 2015, first‐observed patient‐years cannot be interpreted as confirmed first‐ever aesthetic visits. Because a patient‐year is returning by construction whenever the patient was first observed in an earlier year, the proportion of returning patient‐years reflects cohort composition as well as behavioral continuity. We therefore also computed a complementary measure, year‐over‐year retention, defined for each index year as the proportion of patients with a patient‐year in that year who also had a patient‐year in the following calendar year. Retention still depends on observed patient composition and the calendar‐year observation window.

Time to second observed visit was measured in exact fractional days from the first encounter. Patients without a second visit were censored at the end of December 31, 2024, implemented using January 1, 2025 as an exclusive upper bound. Encounters were ordered by patient, timestamp, and original export row order for tied timestamps; records were not reclassified as confirmed treatment episodes. Consecutive billing‐positive intervals were summarized in integer days, excluding zero‐day intervals and using nonoverlapping boundaries of < 90, 90–< 180, 180–< 548, and ≥ 548 days.

Three billing‐based outcomes were prespecified as exploratory: annual total cost, cost per visit, and high‐cost patient‐year. These analyses are reported in their entirety in the Supporting Information. A high‐cost patient‐year is reported using three thresholds: the originally selected absolute threshold of ≥ 10 000 RMB, the top quartile of annual spending, and the top decile of annual spending.

### 2.8 Statistical Analysis

We described annual trends (2015–2024) in total visits and unique patients, and plotted temporal changes in maintenance proportions (≥ 2 and ≥ 3 visits/year). Patient‐origin composition was summarized by the three prespecified periods and visualized at the province level and within‐Beijing district level as descriptive context. For regional comparisons of maintenance utilization, annual visit‐frequency analyses focused on patient‐years from 2022–2024 to reflect the most recent practice phase. Given skewed distributions, continuous variables are reported as medians with interquartile ranges (IQR) and compared using Kruskal–Wallis or Mann–Whitney tests; categorical distributions were compared using chi‐square tests. Crude rank and chi‐square tests are descriptive because patient‐years can repeat within patients; inference for patient‐year associations uses clustered models. All tests were two‐sided. Nominal *p* values and 95% confidence intervals are reported; Benjamini–Hochberg corrections for the three principal interactions and within‐family subgroup contrasts are provided in Table S14.

Multivariable models used Beijing as reference and adjusted for calendar year (categorical), age, and sex. Annual visit count was modeled by Poisson regression, reporting adjusted rate ratios (RR) [29]. Annual total cost was modeled by linear regression of log cost, and high‐cost outcomes by logistic regression. All patient‐year models, including temporal trend and retention models, used standard errors clustered by patient. Cost analyses excluded patient‐years with zero annual cost or any missing charge. For indication adjustment, each outcome was analyzed in three models: the base model in all eligible patient‐years, the same base model in the four‐category complete subset, and the indication‐adjusted model in that identical subset. This separates sample restriction from covariate adjustment. Covariates were retained from the submitted analytic framework rather than selected by statistical significance.

To address the possibility that the temporal increase in maintenance utilization reflected a changing patient mix rather than a change in care patterns, we fitted a logistic model for ≥ 2 visits/year with calendar year as a continuous term, unadjusted and then adjusted for age and sex.

Time to second visit was analyzed using the Kaplan–Meier estimator, compared across origin groups using the log‐rank test, and modeled using Cox proportional‐hazards regression stratified by first‐visit calendar year and adjusted for age and sex. We report the 25th percentile of time to second visit and cumulative return incidence at fixed horizons. A sensitivity analysis was restricted to patients whose first visit occurred on or before June 30, 2023, ensuring at least 18 months of potential follow‐up. For comparison with the conditional analysis reported previously, we also report the observed median interval to the second visit among patients who had at least 2 visits; this quantity conditions on returning and therefore excludes patients who never returned. The proportional‐hazards assumption was examined using scaled Schoenfeld residuals with rank‐transformed time. Because time‐constant associations were not supported for all terms, complementary Cox estimates before and after 180 days are reported in Table S14. Later‐period models use delayed entry at 180 days among patients still at risk. A full‐period HR describes an average association and is not a fixed‐horizon return probability ratio. First‐visit calendar year defines separate baseline‐hazard strata in all Cox models, avoiding unstable coefficients in years with no events and allowing baseline hazards to differ by year. Later‐period HRs are not reported if an origin group has fewer than five events.

Exploratory survival analyses used the strictly observed first‐visit indication proxy, with the same first‐year stratification and age/sex adjustment as the overall model. The origin‐by‐indication interaction was evaluated by likelihood‐ratio comparison of nested Cox models. For patient‐year cadence, the origin‐by‐indication interaction was evaluated by a joint Wald test using patient‐clustered covariance. A patient‐year indication is derived from all recorded diagnoses in that year and may therefore be associated with the opportunity for multiple encounters; this limits interpretation of cadence strata.

Year‐over‐year retention was compared using chi‐square tests and modeled by logistic regression adjusted for index year, age, sex, and index‐year visit count. Because more frequent index‐year attendance might both predict retention and differ by origin, we additionally performed a stratified analysis estimating the origin association separately among patient‐years with 1, 2, and ≥ 3 index‐year visits, a patient‐clustered joint Wald test of the origin × index‐year‐visit‐count interaction, and a model further adjusted for indication proxy. Retention models are reported for index years 2015–2023 pooled and for the recent window 2022–2023. Index year is categorical throughout; index‐year visit count is continuous in the base retention model and categorical in the engagement‐stratified and interaction models. Indication‐adjusted models are paired with a base model in the same complete subset.

Sensitivity analyses included the six‐region grouping, year‐specific and alternative fixed billing thresholds, upper 99th‐percentile winsorization of annual cost, high‐cost thresholds at the 75th and 90th percentiles, and exclusion of billed‐encounter intervals shorter than 30 days. Models estimate associations rather than causal effects. Corrected analyses used Python 3.12 with pandas, NumPy, SciPy, statsmodels, and lifelines; exact versions and the reproducible workflow are documented with Appendix S3. The software is open source. No new clinical instrument, questionnaire, or scale was introduced.

## 3 Results

### 3.1 Service Volume and Patient Characteristics

A total of 48 798 injectable aesthetic dermatology outpatient visits were recorded between 2015 and 2024, in 29 437 unique patients. Annual service volume expanded substantially over the decade (Figure 1). Total visits rose from 3259 in 2015 to 13 700 in 2024, about 4.20‐fold, while annual unique patients rose from 2650 to 9349, about 3.53‐fold. The trajectory was relatively stable during 2015–2020, at roughly 2700–3300 annual visits. It then rose in 2021 (5004 visits; 3837 unique patients). After a transient dip in 2022 (3707; 2810), volume surged in 2023 (8071; 6142) and again in 2024 (13 700; 9349), marking a sharp increase in recorded service activity (Figure 1). Encounter‐level median age increased from 31.1 years in 2015 to 36.5 years (IQR, 30.9–42.8) in 2024 (Table 1).

**FIGURE 1:**
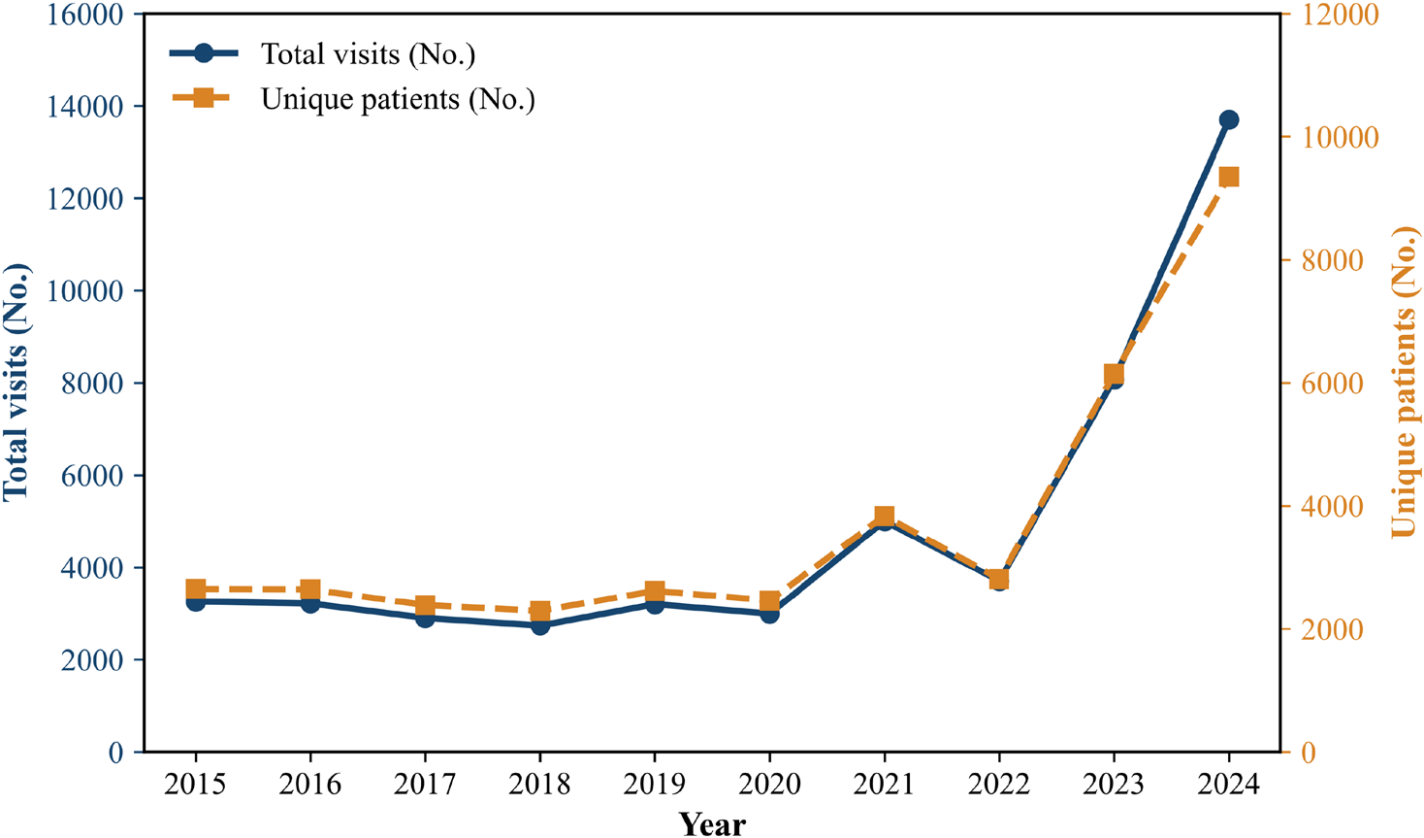
Trends in annual service volume (2015–2024). Dual‐axis line chart showing annual total outpatient visits (left y‐axis) and the number of unique patients (right y‐axis) for injectable aesthetic services from 2015 to 2024. Unique patients were identified by de‐duplicated patient ID within each calendar year.

**TABLE 1:** Overall visit volume and patient characteristics (2015–2024).

Year	Total visits, *n*	Unique patients, *n*	Age, years, median (IQR)	Female, %	≥ 2 visits, %	≥ 3 visits, %
2015	3259	2650	31.1 (26.0–39.2)	94.8	17.6	4.0
2016	3216	2641	31.8 (26.5–40.3)	92.0	16.2	3.7
2017	2905	2386	32.6 (27.1–40.8)	92.2	16.6	3.6
2018	2740	2292	32.7 (27.1–40.4)	91.9	15.0	3.1
2019	3200	2618	33.1 (27.3–41.0)	92.3	16.5	3.4
2020	2996	2461	32.2 (27.3–38.9)	91.3	15.7	3.5
2021	5004	3837	33.3 (28.0–39.6)	91.3	19.8	6.0
2022	3707	2810	35.1 (29.9–41.5)	91.4	20.8	6.3
2023	8071	6142	35.5 (30.0–42.2)	91.5	21.0	6.2
2024	13 700	9349	36.5 (30.9–42.8)	92.0	27.2	9.7

*Note:* Age and female proportion are encounter‐level summaries; age is calculated from date of birth and encounter date, with missing ages excluded from age summaries. Annual unique‐patient counts and ≥ 2/≥ 3 visit percentages count each patient once within a calendar year. Patient‐year age summaries for the maintenance subgroups appear in Table S8.

Abbreviation: IQR, interquartile range.

Patients with and without a resolvable address differed in age, observed return, recorded charges, and first‐visit diagnosis availability (Table S11). Missingness was strongly concentrated in early years: 91.9% of the 4038 patients without a resolvable address first attended in 2015–2017, versus 11.1% of the 25 399 with one. Only 0.40% of patient‐years in 2022–2024 lacked resolvable origin. This reduces the extent of exclusion in recent comparisons but does not establish that between‐group estimates are unbiased. Overall survival estimates apply to the address‐resolvable cohort.

### 3.2 Maintenance Utilization Over Time

Maintenance utilization increased over time (Figure 2). The proportion of patients with ≥ 2 visits within a calendar year increased from 17.6% in 2015 to 27.2% in 2024. Similarly, the proportion of patients with ≥ 3 visits/year rose from 4.0% to 9.7% during the same period (Table 1; Figure 2). These increases were modest before 2021 and became more pronounced thereafter, with the highest proportions observed in 2024 (Figure 2).

**FIGURE 2:**
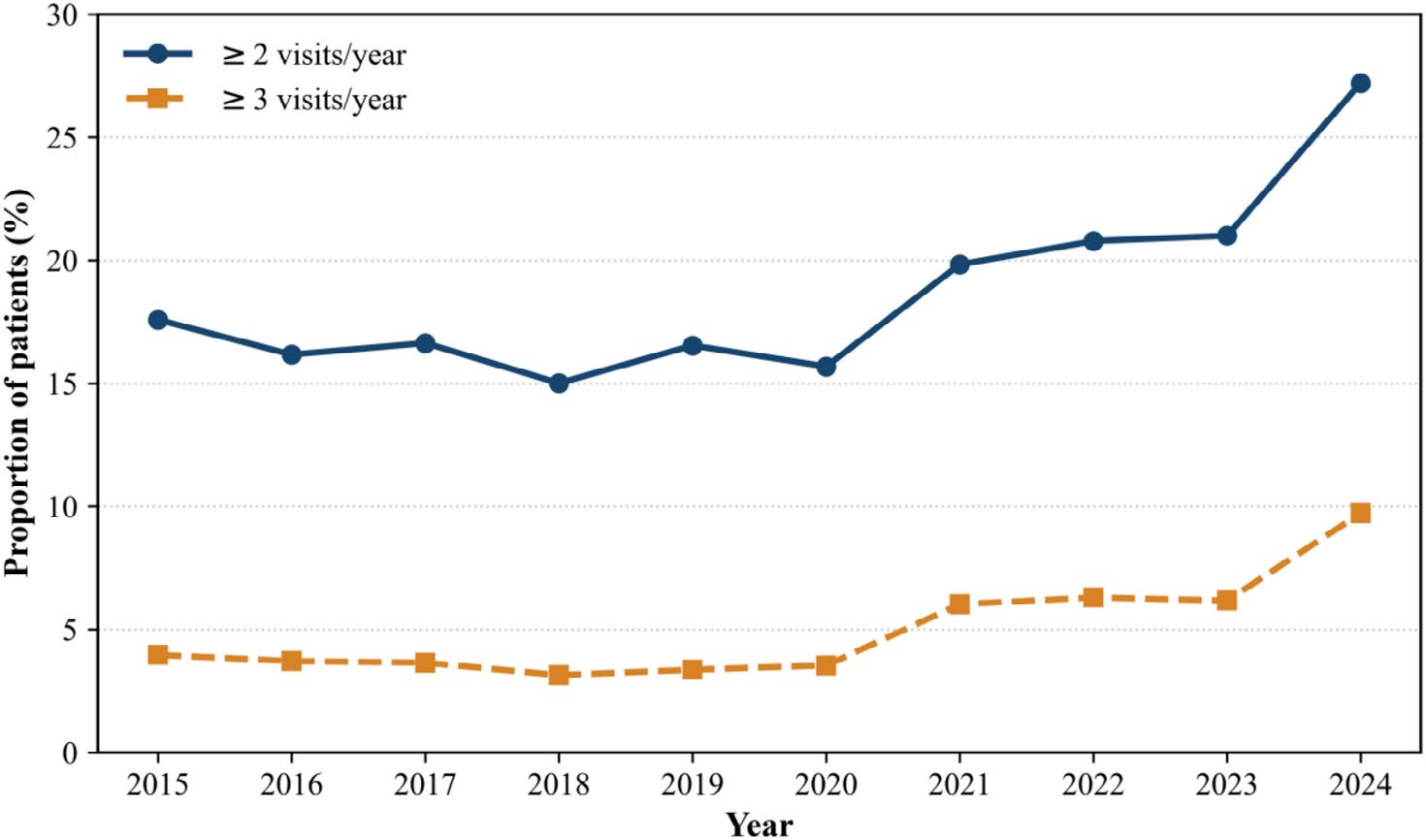
Trends in annual maintenance frequency (2015–2024). Line chart showing the annual proportions of patients with ≥ 2 visits/year and ≥ 3 visits/year. Proportions were calculated using the number of unique patients in each calendar year as the denominator.

Patients attending at least twice annually were slightly older than those attending once, and median age increased in both groups (Table S8). In clustered logistic models, the odds ratio for ≥ 2 visits per calendar year was 1.079 (95% CI 1.068–1.090) before adjustment and 1.074 (95% CI 1.064–1.085) after age and sex adjustment (*p* < 0.001). The adjusted age association was 1.012 (95% CI 1.009–1.015) per year. Age and sex adjustment does not exclude changes in indication mix, service capacity, or available observation time within each calendar year.

### 3.3 Patient‐Origin Composition

Patient‐origin composition changed across the three prespecified periods (Table 2; Figure 3). When data were aggregated into 2015–2019, 2020–2022, and 2023–2024 and categorized into six regions, the share of Beijing residents increased from 65.2% in 2015–2019 to 84.1% in 2020–2022, then decreased to 72.4% in 2023–2024. This pattern is consistent with a contraction of patient‐origin composition toward Beijing during the COVID‐19 pandemic period followed by post‐pandemic re‐expansion (Figure 3). In parallel, the Jing‐Jin‐Ji share decreased during 2020–2022 (5.4%) but rose again to 9.3% in 2023–2024. Other nonlocal regional shares similarly recovered in 2023–2024 (Other Eastern: 5.3%; Central: 4.1%; Western: 5.3%; Northeast: 3.5%) compared with their 2020–2022 lows (2.9%, 2.6%, 3.0%, and 2.1%, respectively). Expressed in the three‐level classification, the distant nonlocal share fell from 25.2% in 2015–2019 to 10.5% in 2020–2022 and recovered to 18.3% in 2023–2024 (Figure 3B).

**TABLE 2:** Geographic distribution of unique patients by region and period (2015–2024).

Region	2015–2019, No. (%)	2020–2022, No. (%)	2023–2024, No. (%)	Total 2015–2024, No. (%)
Beijing	4009 (65.16)	6509 (84.08)	10 058 (72.41)	18 568 (73.11)
Jing‐Jin‐Ji	595 (9.67)	418 (5.40)	1296 (9.33)	2172 (8.55)
Other Eastern	378 (6.14)	224 (2.89)	743 (5.35)	1289 (5.08)
Central	461 (7.49)	202 (2.61)	572 (4.12)	1164 (4.58)
Western	441 (7.17)	228 (2.95)	738 (5.31)	1337 (5.26)
Northeast	269 (4.37)	160 (2.07)	483 (3.48)	869 (3.42)

*Note:* Because a patient may contribute to more than one period, period columns are not additive to the total column. Patients with unresolvable addresses were excluded; the proportion excluded was highest in 2015–2019 and negligible from 2020 onward (Table S11). Jing‐Jin‐Ji denotes Tianjin and Hebei (excluding Beijing).

**FIGURE 3:**
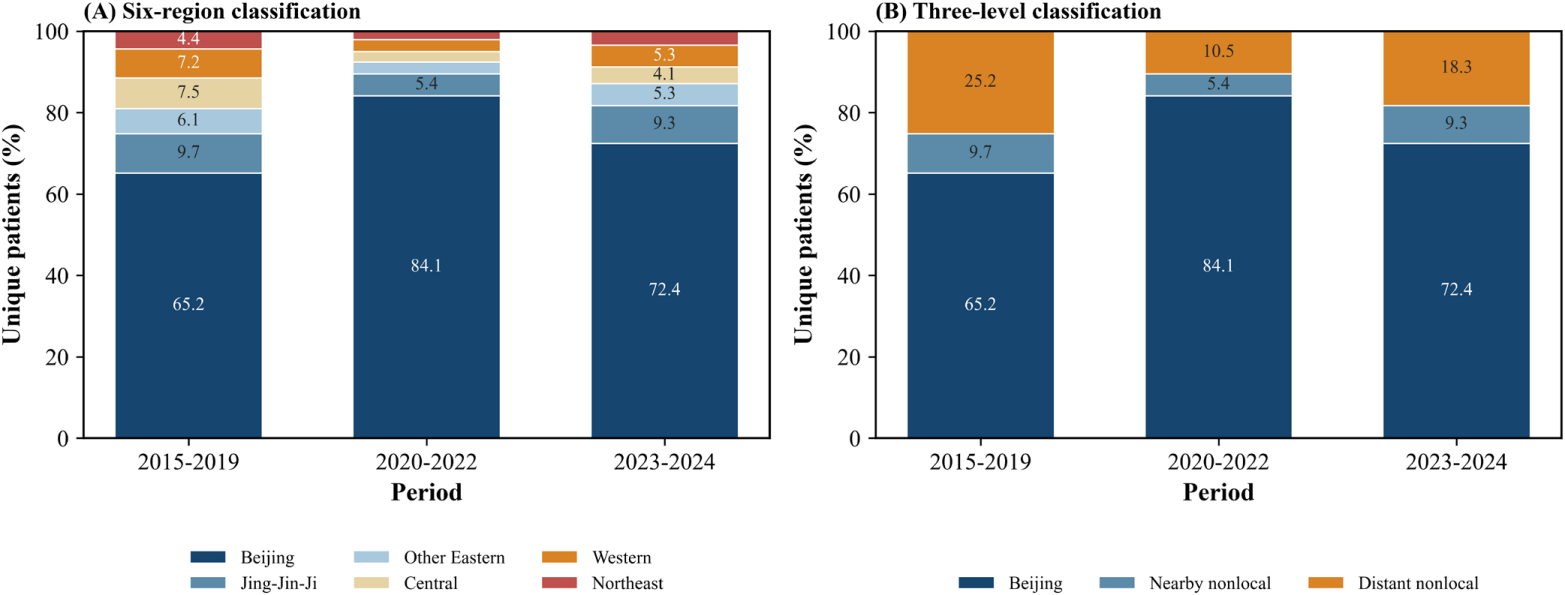
Compositional changes in patient origins by period. One‐hundred‐percent stacked bar plots illustrating the proportional distribution of unique patients across three periods (2015–2019, 2020–2022, and 2023–2024). (A) Six‐region classification: Beijing; Jing‐Jin‐Ji (Tianjin and Hebei); Other Eastern; Central; Western; and Northeast. (B) Three‐level classification used for the primary comparative analyses: Beijing, nearby nonlocal (Tianjin and Hebei), and distant nonlocal (all other non‐Beijing regions). Percentages within each period sum to 100%. Segments below 4% are not labeled.

Detailed province‐level patterns are provided as supplementary context (Figure S1). In brief, the post‐pandemic increase in nonlocal patient origins was most apparent in nearby Jing‐Jin‐Ji sources and selected eastern, western, and northeastern provinces, with Hebei (1009 patients in 2023–2024), Inner Mongolia (419), Shandong (379), and Shanxi (217) contributing the largest nonlocal volumes. Interpreting the rank order of contributing provinces requires caution since it confounds provincial population size, distance from Beijing, local availability of aesthetic services, and unmeasured informational exposure; the pattern is broadly consistent with proximity and population but cannot be attributed to any one of these. These patterns should be interpreted as descriptive patient‐origin composition rather than direct evidence of travel burden, referral pathways, or spatial accessibility.

Within Beijing, district‐level patterns were also treated as supplementary descriptive context (Figure S2; plotting code in Appendix S4). Patient origins remained concentrated in central urban districts, with outward growth in selected suburban districts during 2023–2024. These findings are compatible with residential density, transport networks, and local aesthetic‐service availability, none of which were measured here, and the outward growth in 2023–2024 coincided with the overall expansion in service volume rather than being independent of it.

### 3.4 Diagnosis‐Derived Indication Proxy Mix

Among the 21 315 encounters in 2022–2024 with a recorded primary diagnosis (83.7% of encounters in that window), the indication proxy mix was 38.3% neuromodulator‐type, 27.7% volume/contour‐type, 24.4% skin‐quality‐type, 8.1% post‐procedure review, and 1.5% other or unclassifiable. Median visit‐level charges were broadly consistent with these categories (neuromodulator‐type 5202 RMB, IQR 3787–10 156; volume/contour‐type 6471 RMB, IQR 60–12 699; skin‐quality‐type 6282 RMB, IQR 3432–10 252; post‐procedure review 648 RMB, IQR 174–952), supporting the face validity of the mapping without confirming the products or procedures used.

At the patient‐year level, 15 665 of 18 227 patient‐years (85.9%) could be assigned an indication proxy. The distribution differed across origin groups (*p* < 0.001): neuromodulator‐type indications accounted for 45.6% of Beijing patient‐years, 40.1% of nearby nonlocal patient‐years, and 42.3% of distant nonlocal patient‐years; volume/contour‐type indications for 25.6%, 30.2%, and 31.3%, respectively; and skin‐quality‐type indications for 23.8%, 17.4%, and 15.7%, respectively (Table S2). Nonlocal patient‐years also included a higher proportion of post‐procedure review encounters (10.3% and 9.4% vs. 4.0%). Six‐region proportions are provided in Table S2. As noted in the Methods, these proportions describe the dominant recorded indication under a fixed hierarchical rule and cannot be read as the prevalence of treatment types.

### 3.5 Annual Maintenance Cadence by Patient Origin

In the patient‐year analyses from 2022 to 2024 (*n* = 18 227 patient‐years with a resolved origin), annual maintenance cadence was low and similar across origin groups (Table 3; Figure 4). The median annual visit count was 1 visit per year (IQR, 1–1) in all three groups, and the distribution across 1, 2, and ≥ 3 visits did not differ significantly (Kruskal–Wallis *p* = 0.173; Pearson chi‐square *p* = 0.150). The great majority of patient‐years comprised a single visit in every group: 75.5% for Beijing, 77.2% for nearby nonlocal, and 76.8% for distant nonlocal patient‐years. The proportions of patient‐years with ≥ 2 visits were correspondingly 24.5%, 22.8%, and 23.2%. The six‐region classification gave consistent results (Kruskal–Wallis *p* = 0.265; chi‐square *p* = 0.104; Table S1; Figure S3).

**TABLE 3:** Annual visit frequency and maintenance cadence by patient origin, three‐level classification (2022–2024).

Patient origin	*N* (patient‐years)	Visits, median (IQR)	1 visit, No. (%)	2 visits, No. (%)	≥ 3 visits, No. (%)	≥ 2 visits (%)
Beijing	13 775	1 (1–1)	10 404 (75.5)	2253 (16.4)	1118 (8.1)	24.5
Nearby nonlocal	1507	1 (1–1)	1163 (77.2)	218 (14.5)	126 (8.4)	22.8
Distant nonlocal	2945	1 (1–1)	2261 (76.8)	472 (16.0)	212 (7.2)	23.2
Overall *p* value		0.173 (a)		0.150 (b)		

*Note:* Nearby nonlocal denotes Tianjin and Hebei; distant nonlocal denotes all other non‐Beijing regions. (a) Kruskal–Wallis test comparing the distribution of annual visit counts across the three origin groups. (b) Pearson chi‐square test comparing the proportions of visit‐frequency categories (1, 2, and ≥ 3 visits) across the three origin groups. These crude tests did not detect clear distribution differences; they do not establish equivalence and do not account for repeated patient‐years. Clustered adjusted estimates appear in Table 4. The corresponding six‐region analysis is presented in Table S1, and indication‐stratified models in Table S12.

Abbreviation: IQR, interquartile range.

**FIGURE 4:**
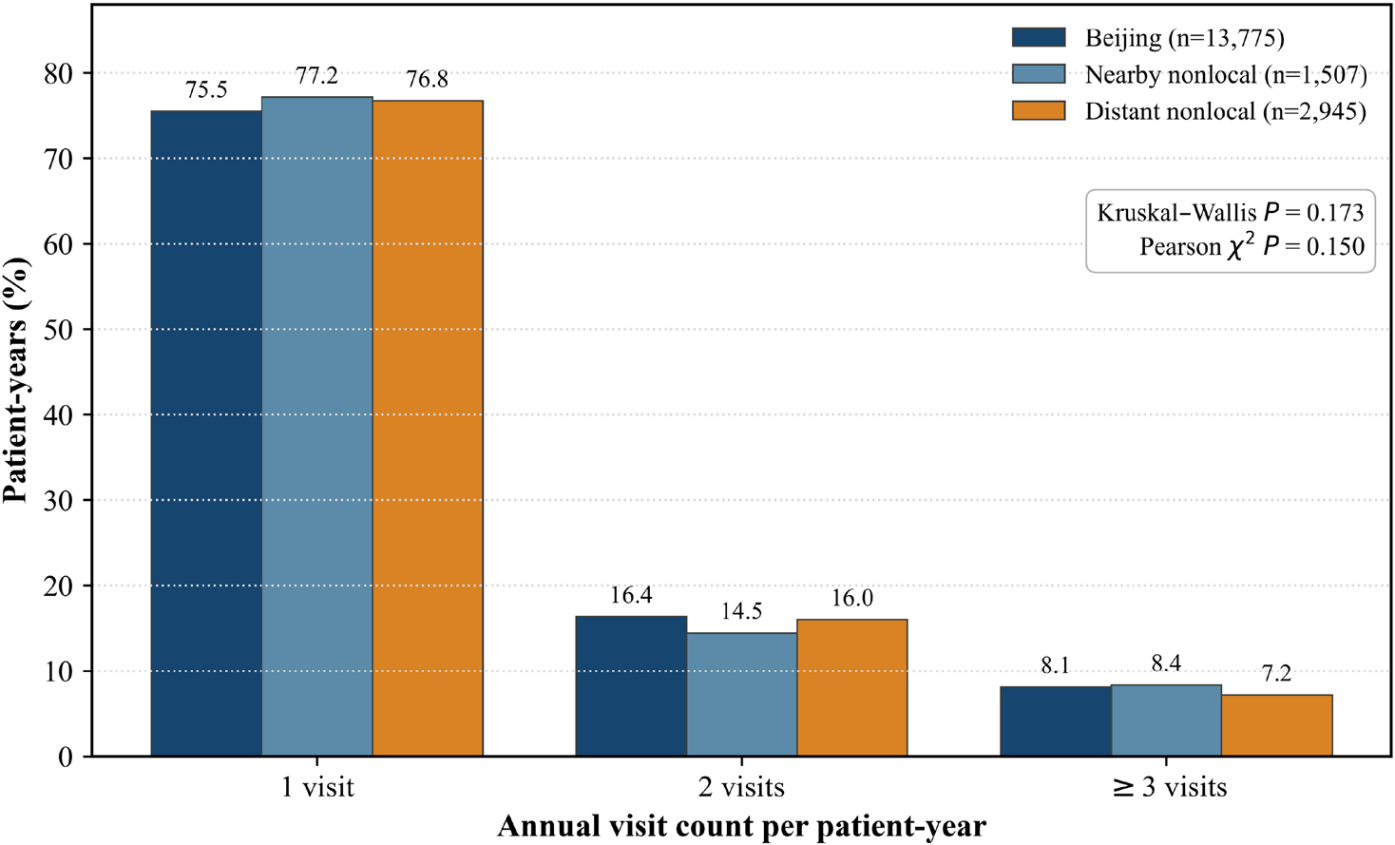
Distribution of annual visit counts by patient origin (2022–2024). Grouped bar chart showing the percentage of patient‐years with 1, 2, and ≥ 3 visits within a calendar year for each origin group, with the corresponding overall test statistics. The equivalent violin‐plot representation is provided as Figure S3.

In patient‐clustered Poisson models, nearby nonlocal origin had an adjusted RR of 0.994 (95% CI 0.953–1.036) (*p* = 0.772), and distant nonlocal origin an RR of 0.957 (95% CI 0.931–0.983) (*p* = 0.001; Table 4). The corresponding six‐region estimates are provided in Table S3. These relative differences were small; no equivalence margin or minimum clinically important difference was specified.

**TABLE 4:** Multivariable Poisson model for annual visit count, three‐level classification (2022–2024).

Origin (vs Beijing)	Adjusted RR (95% CI)	*p*
Nearby nonlocal	0.994 (0.953–1.036)	0.772
Distant nonlocal	0.957 (0.931–0.983)	0.001

*Note:* Annual visit count was modeled using Poisson regression with standard errors clustered by patient and is reported as adjusted rate ratios. The model was adjusted for calendar year (categorical: 2022/2023/2024), age, and sex. Reference category: Beijing. Unit of analysis: patient‐year. *N* = 18 227. The corresponding six‐region model is presented in Table S3, indication‐stratified models and the origin × indication interaction test in Table S12, and models for annual total cost in Table S7.

The origin‐by‐indication interaction for annual count had a patient‐clustered Wald *χ* ^2^ = 17.31, df = 6, *p* = 0.008 (BH‐adjusted *p* = 0.012). Stratum‐specific estimates are reported in Table S12. For distant nonlocal origin, adjusted RRs were 0.94 (95% CI 0.90–0.98), 0.93 (95% CI 0.88–0.98), and 0.94 (95% CI 0.89–1.00) in the first three categories, respectively. For nearby nonlocal origin in the post‐procedure review stratum, the RR was 1.37 (95% CI 1.13–1.65). These analyses describe a diagnosis hierarchy derived across the patient‐year and cannot identify treatment‐specific causal effects.

### 3.6 Continuity of Care: Returning Patient‐Years and Year‐Over‐Year Retention

During 2022–2024, returning patient‐years accounted for 23.5% of Beijing patient‐years, compared with 14.4% among nearby nonlocal and 13.7% among distant nonlocal patient‐years. Among all returning patient‐years, 45.6% belonged to patients first observed 1 year earlier, whereas 38.9% belonged to patients first observed at least 3 years earlier and 6.3% to patients first observed in 2015–2016; 25.0% represented re‐presentation after at least one full calendar year of absence. Consistent with the cumulative definition, all 85 patients who were first observed in 2015 and attended again in 2024 were classified as returning in 2024.

Because a patient‐year is returned by construction when the patient was first observed earlier, this contrast partly reflects cohort composition: 70.0% of Beijing patient‐years in 2022–2024 belonged to patients first observed in 2023–2024, compared with 86.5% of nearby nonlocal and 87.4% of distant nonlocal patient‐years. In other words, the nonlocal re‐expansion consisted largely of newly observed patients.

Year‐over‐year retention reduces the cumulative‐history dependence of returning‐patient‐year status but remains composition‐dependent. For index years 2022–2023, retention was 26.0%, 21.9%, and 21.2% in Beijing, nearby nonlocal, and distant nonlocal patient‐years; pooled 2015–2023 values were 25.5%, 20.4%, and 17.9% (Table S4). Models adjusted for categorical index year, age, sex, and continuous index‐year visit count used patient‐clustered standard errors. Recent‐window adjusted ORs were 0.82 (95% CI 0.65–1.04) and 0.76 (95% CI 0.65–0.89) for nearby and distant nonlocal origin; pooled estimates were 0.75 (95% CI 0.64–0.87) and 0.63 (95% CI 0.56–0.71), respectively.

Retention increased with index‐year visit count in every origin group. In the pooled main‐effects model, adjusted ORs versus one visit were 3.27 (95% CI 3.02–3.55) for two visits and 6.34 (95% CI 5.60–7.17) for three or more. Crude retention percentages for the 1‐, 2‐, and ≥ 3‐visit strata were 19.3%, 45.0%, and 61.2% in Beijing; 17.1%, 33.2%, and 44.2% in nearby nonlocal; and 13.9%, 33.5%, and 50.9% in distant nonlocal patient‐years. The origin‐by‐count interaction had a patient‐clustered Wald *χ* ^2^ = 9.00, df = 4, *p* = 0.061 (BH‐adjusted *p* = 0.061). Separate estimates and same‐subset indication‐adjusted comparisons are reported in Table S13. These associations do not show that increasing visits would itself cause improved retention.

### 3.7 Time to Second Visit

Among 25 399 patients with resolvable origin, 7565 (29.8%) had a second observed visit (Table 5). Cumulative return estimates and the 25th percentile with 95% confidence intervals are reported in Table 5; Kaplan–Meier curves appear in Figure 5. The observed time to second visit or administrative censoring had a median of 421 days (IQR 131–1318); median potential observation from first visit to study end was 811 days. Full‐period adjusted HRs were 0.94 (95% CI 0.86–1.02) for nearby and 0.83 (95% CI 0.78–0.88) for distant nonlocal origin. The corresponding distant nonlocal HR was 0.74 (95% CI 0.69–0.81) among 15 174 patients first observed before July 2023. Proportional‐hazards diagnostics and separate 0–180‐day and later estimates are reported in Table S14; full‐period HRs should not be interpreted as constant effects throughout follow‐up. Median follow‐up estimated by reverse Kaplan–Meier was 922 days.

**TABLE 5:** Time to second observed visit by patient origin (2015–2024).

Patient origin	*N*	Second visit, No. (%)	25th percentile, days (95% CI)	Cumulative return, % (180/365/1095 days)	Adjusted HR (95% CI)	*p*
Beijing	18 568	5752 (31.0)	287 (269–303)	18.5/27.8/34.7	Reference	—
Nearby nonlocal	2172	608 (28.0)	316 (248–380)	18.5/26.4/32.9	0.94 (0.86–1.02)	0.118
Distant nonlocal	4659	1205 (25.9)	411 (343–514)	17.3/24.2/30.0	0.83 (0.78–0.88)	< 0.001

*Note:* Time was measured in fractional days from the first observed encounter through the end of December 31, 2024. The 25th percentile CI was obtained by inversion of the Greenwood log–log Kaplan–Meier confidence bands. Log‐rank *p* < 0.001. Cox models stratify baseline hazards by first‐visit year and adjust for age and sex. An HR is a relative instantaneous event rate, not a probability ratio. Time‐specific models and horizon‐specific CIs are provided in Table S14. Analyses are restricted to patients with resolvable origin; corresponding curves are in Figure 5.

Abbreviations: CI, confidence interval; HR, hazard ratio; NR, not reached.

**FIGURE 5:**
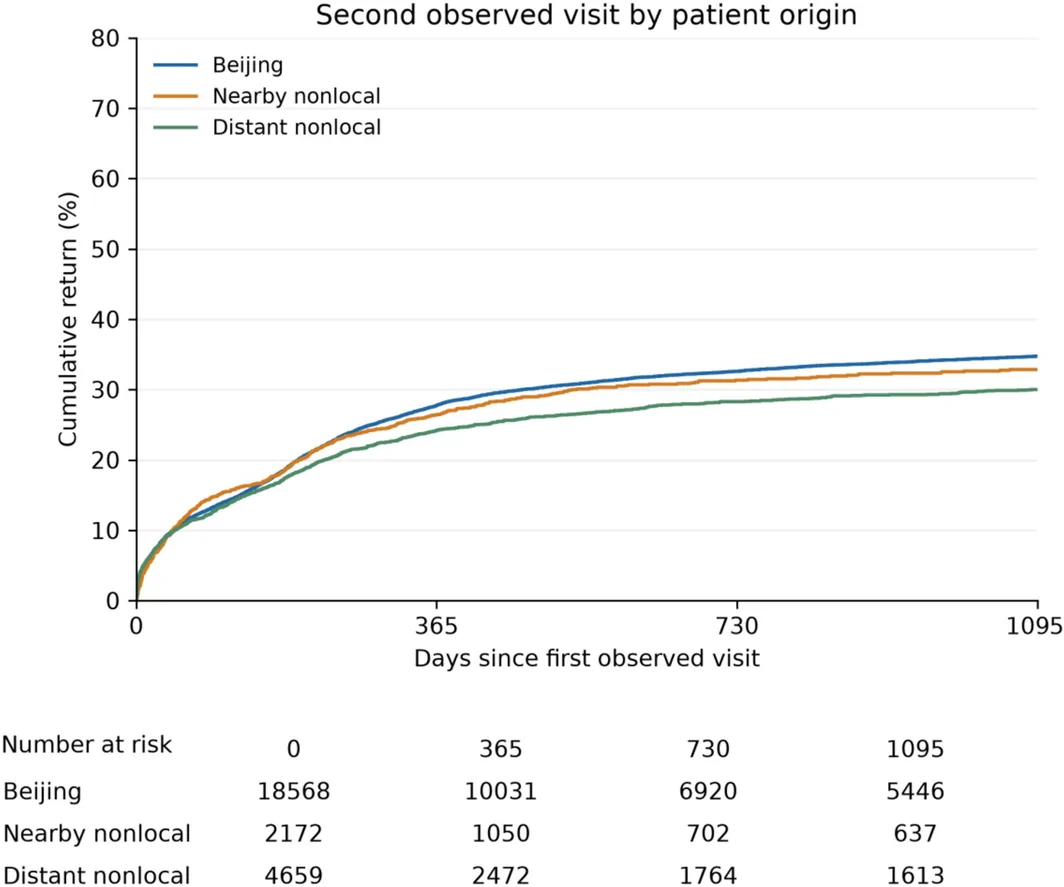
Kaplan–Meier estimate of cumulative return to a second observed visit by administrative origin. All first encounters through December 31, 2024 are included; censoring occurs at the end of that day. Curves are displayed through 1095 days, with numbers at risk below. Administrative non‐return does not establish discontinued care. See Table 5 and Table S14.

By contrast, the analysis restricted to patients who did return, which conditions on a second visit, reproduced the shorter intervals reported previously: median time to second visit was 144.6 days for Beijing patients, 91.0 days for nearby nonlocal patients, and 107.2 days for distant nonlocal patients, with 56.9%, 61.8%, and 62.8% returning within 180 days. These two analyses answer different questions: the origin groups differed in observed re‐engagement, while the conditional interval comparison was restricted to those with a documented return and cannot describe patients who had not returned.

### 3.8 Time to Second Visit Stratified by Indication Proxy

Among 17 008 patients whose first encounter carried a diagnosis in one of the four main proxy categories, 5418 had a second visit. First‐encounter diagnosis was available for 17 184 of 25 399 patients (67.7%); missing first diagnoses were not filled from later encounters. The origin‐by‐indication interaction had likelihood‐ratio *χ* ^2^ = 35.78, df = 6, *p* < 0.001 (BH‐adjusted *p* < 0.001; Table 6).

**TABLE 6:** Time to second observed visit by patient origin, stratified by diagnosis‐derived indication proxy of the first visit (2015–2024).

First‐visit indication proxy	Patient origin	*N*	Second visit, No. (%)	Cumulative return, % (365/1095 days)	Adjusted HR (95% CI)	*p*
Neuromodulator‐type	Beijing	5246	1767 (33.7)	33.6/43.9	Reference	—
	Nearby nonlocal	552	144 (26.1)	28.5/37.0	0.79 (0.67–0.94)	0.007
	Distant nonlocal	1226	355 (29.0)	30.0/39.0	0.86 (0.77–0.97)	0.011
Volume/contour‐type	Beijing	2359	587 (24.9)	30.9/46.3	Reference	—
	Nearby nonlocal	336	68 (20.2)	25.5/NE	0.88 (0.68–1.13)	0.319
	Distant nonlocal	723	155 (21.4)	28.2/55.2	0.90 (0.76–1.08)	0.269
Skin‐quality‐type	Beijing	4360	1571 (36.0)	28.6/37.6	Reference	—
	Nearby nonlocal	460	168 (36.5)	30.1/39.8	1.00 (0.85–1.18)	0.987
	Distant nonlocal	881	265 (30.1)	24.2/32.4	0.75 (0.66–0.86)	< 0.001
Post‐procedure review	Beijing	470	162 (34.5)	37.2/NE	Reference	—
	Nearby nonlocal	138	62 (44.9)	49.7/NE	1.47 (1.10–1.98)	0.010
	Distant nonlocal	257	114 (44.4)	47.3/NE	1.38 (1.09–1.76)	0.009

*Note:* Strata use the first encounter row only, preserving missing diagnosis. The four categories include 17 008 patients and 5418 s visits. Within‐stratum Cox models stratify baseline hazards by first‐visit year and adjust for age and sex; Beijing is reference. The standard log‐rank *p* values are 0.005, 0.305, 0.002, 0.004, respectively. The origin‐by‐indication interaction has likelihood‐ratio *χ* ^2^ = 35.78, df = 6, *p* < 0.001. Proxies are unvalidated; full‐period HRs, late‐tail probabilities, and subgroup comparisons require caution. See Table S14 for diagnostics, CIs, and multiplicity checks. NE denotes a probability horizon beyond observed follow‐up support in that group; these values are not extrapolated.

Abbreviations: CI, confidence interval; HR, hazard ratio.

Adjusted HRs for distant nonlocal origin were 0.86 (95% CI 0.77–0.97) for neuromodulator‐type, 0.90 (95% CI 0.76–1.08) for volume/contour‐type, and 0.75 (95% CI 0.66–0.86) for skin‐quality‐type first visits. In the post‐procedure review stratum, the nearby and distant nonlocal estimates were 1.47 (95% CI 1.10–1.98) and 1.38 (95% CI 1.09–1.76), respectively. The complete stratum estimates appear in Table 6 and Figure 6, with multiplicity and time‐dependence checks in Table S14. The proxies do not confirm procedures administered or the location of any preceding treatment; these analyses are exploratory.

**FIGURE 6:**
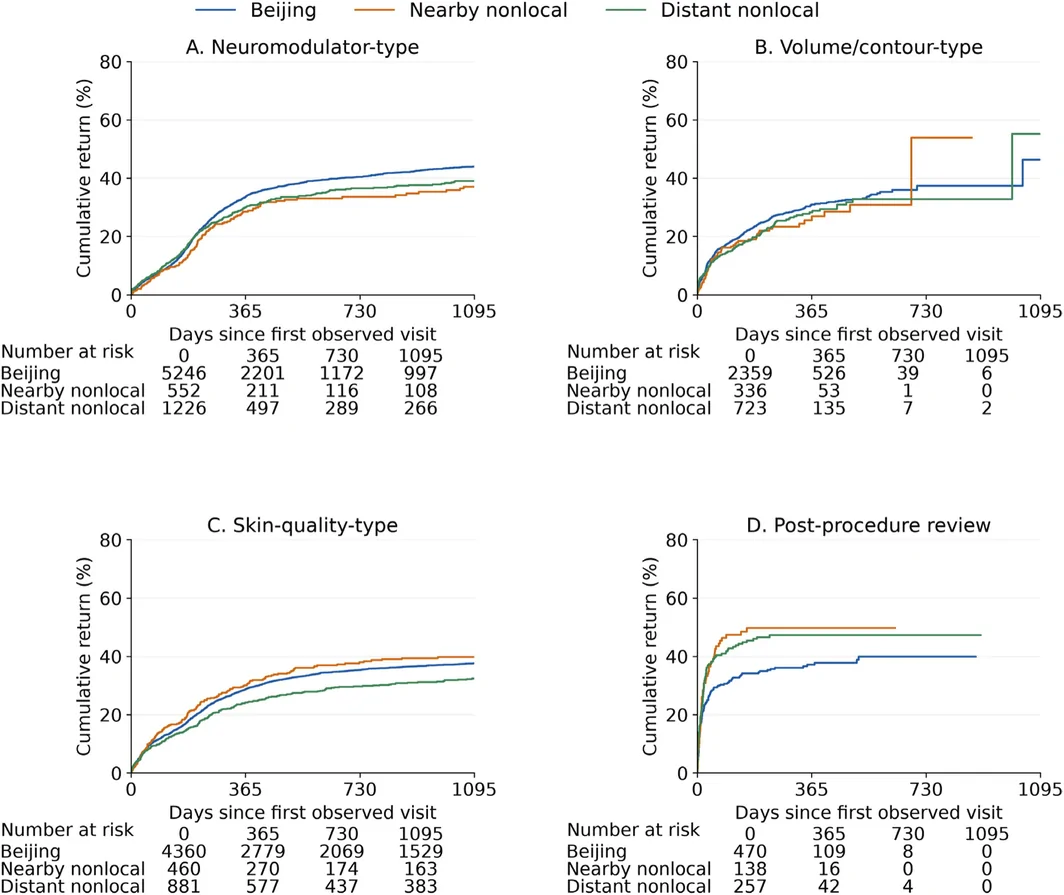
Kaplan–Meier estimate of cumulative return by strictly recorded first‐visit indication proxy and administrative origin. Panels show (A) neuromodulator‐type, (B) volume/contour‐type, (C) skin‐quality‐type, and (D) post‐procedure review. Missing first diagnoses are not backfilled from later visits. Censoring is at the end of December 31, 2024, and curves are displayed through 1095 days with risk counts. Sparse late risk sets warrant caution. Estimates and diagnostic checks appear in Table 6 and Table S14.

### 3.9 Treatment Visits and Observed Billed‐Encounter Intervals

Across all recorded encounters, 33.8% carried a charge at or below the 60 RMB threshold and were classified as consultation‐only visits. The annual proportion of treatment visits ranged from 57.0% in 2021 to 70.3% in 2023. The sensitivity analyses indicate that this variation partly depends on the billing threshold. The modal low charge was below 60 RMB in most years, so a fixed threshold classifies some low‐cost billed encounters as consultation‐only and does so unevenly across years. Applying the year‐specific modal fee instead, the treatment‐visit proportion rose from 66.1% to 73.0% overall. The largest single‐year change was in 2016, from 61.0% to 80.1%, the year with the lowest empirical low‐charge mode. The ranking of years is itself threshold‐dependent. The 2021 minimum persisted under fixed thresholds of 60, 100, and 200 RMB but not under the year‐specific fee, where 2015 was lowest (Table S10). The year‐to‐year series should therefore not be read as a trend in practice. There were 65 encounters with missing charges, which remained unclassified.

Annual treatment‐visit counts in 2022–2024 were low and did not differ across origin groups (median 1, IQR 0–1 in all groups; ≥ 2 treatment visits in 15.9%, 15.9%, and 15.7%; Kruskal–Wallis *p* = 0.936).

There were 13 240 positive intervals between consecutive encounters billed above 60 RMB; 34 zero‐day intervals were excluded. The median was 187 days (IQR 84–310). Categorizing intervals by the diagnosis at the ending encounter, medians were 224 days (IQR 156–342) for neuromodulator‐type, 105 days (IQR 36–220) for volume/contour‐type, 194 days (IQR 102–325) for skin‐quality‐type. The preceding encounter could have a different or missing indication. After excluding intervals shorter than 30 days, the respective medians were 230, 144, 209 days. Among intervals ending in a neuromodulator‐type indication, 11.5% were < 90 days, 21.1% were 90–< 180 days, 56.9% were 180–< 548 days, and 10.5% were ≥ 548 days; 67.4% were ≥ 180 days. Small rounding differences may affect the sum of percentages.

The interval summaries varied with the billing threshold, although the neuromodulator‐type median remained longer than 6 months under each definition (Table S10). With the year‐specific modal fee, the overall and neuromodulator‐type medians were 176 and 219 days, with 20.9% of neuromodulator‐type intervals at 90–< 180 days. Threshold sensitivity does not validate these as same‐product retreatment intervals.

### 3.10 Exploratory Billing‐Based Outcomes

Exploratory billing analyses excluded 38 recent patient‐years with missing charges and 8 complete patient‐years with zero total cost, leaving 18 181. Base‐model adjusted cost ratios were 0.92 (95% CI 0.81–1.05) and 0.96 (95% CI 0.87–1.06) for nearby and distant nonlocal origin. The top‐quartile and top‐decile thresholds were 11 557 and 19401.5 RMB. Descriptive percentages and clustered logistic models at all three thresholds appear in Tables S6 and S7 and Figure S4.

In the identical 15 457‐patient‐year four‐category subset, the distant nonlocal adjusted cost ratio was 1.01 (95% CI 0.92–1.11) before and 1.15 (95% CI 1.05–1.25) after indication adjustment; the corresponding ≥ 10 000‐RMB ORs were 1.18 (95% CI 1.07–1.29) and 1.29 (95% CI 1.17–1.42). Sample restriction and indication adjustment are thus reported separately. These findings do not establish a mechanism: the indication proxy is incomplete and unvalidated, and treatment mix, doses, product prices, package billing, and physician factors were unavailable. Charges cannot be interpreted as treatment intensity, product selection, clinical outcomes, or preference.

## 4 Discussion

In this 10‐year study, recorded service volume and repeat annual attendance increased, while single‐visit patient‐years remained common. Recent annual count differences across administrative origin groups were small. Time‐to‐second‐visit and next‐year retention analyses suggested modest differences in observed re‐engagement at this center. Exploratory first‐visit indication strata showed heterogeneity, with uncertainty and time dependence that limit a single mechanistic explanation.

### 4.1 Observed Attendance in Relation to Expected Retreatment Intervals

Published product‐duration evidence provides clinical context, but these data do not identify administered products or planned retreatment. Botulinum toxin type A effects in facial aesthetic use commonly last approximately 3–4 months [3], while many efficacy trials follow a single cycle [7]. Intervals ending in a neuromodulator‐type diagnosis had a median of about 7.4 months in this dataset, and roughly one‐fifth were 90–< 180 days. This describes billed encounter spacing at one center, not adherence to a verified neuromodulator regimen. For volume/contour‐type diagnoses, the shorter interval may include staged procedures, reviews, different modalities, or early refinement. MRI persistence of hyaluronic acid material does not establish the duration of a satisfactory clinical effect or an appropriate retreatment schedule [8, 9]. Excluding short intervals necessarily increases the median and does not independently confirm staged treatment.

Patient preference for intermittent correction and constraints such as cost, work, travel, and appointment availability are plausible explanations for observed attendance. Other explanations include treatment at another institution, a different treatment modality, or a billing record that does not capture the procedure. These possibilities cannot be separated without procedure‐level and patient‐reported information. The observed interval distribution should therefore motivate further study rather than define a clinical maintenance standard.

The observed increase in maintenance utilization persisted after age and sex adjustment. Patients attending at least twice per year were slightly older, and the cohort aged over the decade, but the calendar‐year trend in ≥ 2 visits/year was essentially unchanged after adjustment for age and sex (adjusted OR 1.074 vs. unadjusted 1.079 per year). This is compatible with more frequent recorded attendance but does not exclude changes in unmeasured case mix or service capacity, and it is compatible with broader adoption of combination and repeat treatment strategies [25, 26, 27] and an increasing emphasis on skin quality as a treatment target [30]. Treatment‐planning behavior was not directly measured; however, a parallel shift in the case mix presenting to the service, rather than in how any given patient is managed, would produce the same aggregate pattern.

### 4.2 Continuity, Geographic Origin, and the Value of a Time‐To‐Event Approach

The conditional comparison among patients who returned and the time‐to‐event analysis address different questions. The latter retains people without a documented second visit through administrative censoring and estimates return over time. Distant nonlocal origin had a lower full‐period second‐visit hazard (adjusted HR 0.83), but the proportional‐hazards diagnostics indicate that a constant association over follow‐up is an approximation. Neither analysis establishes whether patients who did not return discontinued care, transferred elsewhere, or simply had a longer intended interval.

Next‐year retention uses a common calendar‐year horizon and reduces the cumulative‐history dependence of returning‐patient‐year status. It does not remove differences in patient mix or opportunity to attend. The paired models in Table S13 show how origin estimates change with sample restriction and indication adjustment.

First, the gradient is modest in absolute terms. It amounts to roughly 4 to 5 percentage points in the recent window, against a background in which about three‐quarters of patients in every group did not return the following year.

Index‐year attendance had a larger association with next‐year return than origin in the fitted model. However, this may reflect stable patient preferences, ongoing treatment episodes, or other unmeasured factors. The stratified estimates and interaction test quantify variation across engagement levels without establishing equivalence between strata or the benefit of an intervention that increases visit frequency.

Studies in other clinical settings report that greater travel distance and travel time are associated with lower persistence of treatment [31], and that travel burden to procedural dermatologic care falls unevenly across populations [32]. Travel time has likewise been linked to health care costs and resource use [33]. A genuine continuity gradient is therefore plausible. Distance itself, however, was not measured here.

### 4.3 Heterogeneity by First‐Visit Indication Proxy

The corrected first‐encounter analysis describes heterogeneity across diagnosis proxies (Table 6). A diagnosis recorded at a later encounter was not allowed to define baseline, because that would use information available only after a patient had returned. The observed differences can generate hypotheses about indication mix and continuity, but do not identify a geographic mechanism.

Availability of local alternatives and arrangements for follow‐up could influence return patterns. Neither local service availability nor a prearranged appointment was recorded. A first observed post‐procedure review diagnosis also does not establish that the preceding treatment was performed at this center. These explanations therefore remain hypotheses.

Nonlocal patient‐years contained proportionally more post‐procedure review and volume/contour‐type diagnoses. This could contribute to conditional interval differences, but the present categories cannot distinguish scheduled review from elective retreatment or explain the shorter intervals causally.

The post‐procedure review stratum contained 865 patients with complete covariates. Sparse late risk sets and possible differences in diagnostic recording limit interpretation, particularly at 1095 days. The subgroup confidence intervals, multiplicity‐adjusted *p* values, and time‐specific estimates should be considered together; the data do not establish that geographic distance selectively impairs elective care.

### 4.4 What Geographic Origin Does and Does Not Indicate

Although geographic origin has been used in broader health services research to understand patient catchments and specialty‐care utilization [10, 11, 13, 34, 35, 36], our findings must be interpreted cautiously. Most importantly, the similarity of annual visit cadence across origin groups must not be read as evidence that geographic barriers to care are absent. Patients recorded in these data are, by definition, patients who reached the center at least once; any barrier that prevents attendance entirely is invisible to this design, and the lower return probability observed among distant patients is itself compatible with the presence of such barriers. Travel distance, travel time, transportation mode, out‐of‐pocket burden, income, and educational attainment were not measured, and no inference about equity of access can be drawn. In this setting, patient origin may be most useful as a pragmatic descriptor for planning communication, scheduling, and continuity pathways rather than as a measure of access barriers [37]. Nonlocal patients might consolidate care into fewer, more comprehensive encounters, but products, doses, combination procedures, and package billing were unavailable, so this explanation cannot be tested here.

No patient had more than one distinct recorded address in the available extract. This limits reconstruction of historical residence, but does not establish whether the source system retained or overwrote historical addresses. The direction and magnitude of bias from unobserved relocation cannot be determined.

The three‐level classification offers a simpler primary comparison, with the six‐region analysis as a sensitivity check. Crude distribution tests did not detect clear differences; adjusted associations were small. A nonsignificant test does not establish equivalence, and relative differences should be interpreted with absolute attendance distributions and their uncertainty.

### 4.5 Interpreting the Setting: A Single Public Tertiary Center

The most consequential limitation of this study is its single‐center design, and we set out what it does and does not permit.

The center is a public, hospital‐based tertiary academic specialty service in Beijing. Three features of that setting should be assumed not to transfer. First, case mix and price: a hospital‐based service in a capital city, with fees set within a public system, will differ from private clinics and commercial chains in the proportion of complex or revision work, in price level, and in the mix of neuromodulator, filler, and device‐based treatment. Second, patient composition: the encounter‐level median ages of 31.1–36.5 years, the 91% to 95% female proportion, and the very high Beijing share are properties of this catchment. Third, the meaning of “distant”: in a country the size of China this spans travel times from a few hours to a full day, and the same label in a smaller country, or in a multi‐site group with regional branches, would describe an entirely different burden.

Both the direction and the magnitude of these findings require external validation. Prices, case mix, recall systems, service capacity, and incomplete capture of care elsewhere may influence second‐visit rates, annual cadence, and billed encounter intervals. The present values should not be used as reference standards for other practices.

Two contrasts illustrate the point. In a private practice with active recall systems, retention would plausibly be higher and the geographic gradient narrower. In a multi‐center group offering follow‐up at a branch nearer the patient's home, the post‐procedure review pattern reported here might disappear altogether, because the reason to return to *this* center would be removed. Multi‐center studies spanning public and private settings and several regions are needed before any of these quantities can be treated as generalizable. We make no claim that they are.

The absence of a structured referral pathway does not rule out informal professional referrals, personal recommendations, or online reputation. These factors could influence catchment composition and return patterns and were not measured. Evidence on social‐media influence and patient motivations provides context, but does not explain individual behavior in this cohort [38, 39].

### 4.6 Service‐Level Changes During the Study Period

Several service‐level and external changes may have influenced the observed trends. The empirical annual low‐charge mode ranged from 14 to 60 RMB, and no inflation adjustment was applied; this primarily affects classification of treatment visits, as shown in the threshold sensitivity analysis. From 2023, increased clinical staffing and online patient‐facing activity coincided with the sharp rise in service volume and the recovery of nonlocal patient share. These changes cannot be separated from deferred post‐pandemic demand, and the recovery in geographic composition therefore cannot be attributed to travel restrictions alone. No audited record was available to quantify changes in service lines, staffing, referral activity, or marketing. More broadly, changes in the medical‐aesthetics market and regulatory environment occurred during the study period but were not measured, so no observed trend can be attributed to any one external factor.

### 4.7 Practical Implications

These findings generate three practice hypotheses rather than validated intervention recommendations. First, because index‐year engagement was associated with subsequent retention, practices could test whether completing treatment planning and offering a follow‐up appointment at the initial visit improves second‐visit conversion, particularly for patients who travel from outside the region. Second, the observed interval distribution may inform, but cannot establish, the timing of reminder interventions; prospective studies should compare reminder schedules without deriving an optimal clinical interval from these administrative data. Third, written expectations about the likely retreatment window, a clear contact route for concerns, and remote triage may be especially useful for patients returning to distant locations. Patient‐reported intent and barriers should be collected to distinguish episodic preference from constrained access. Reports of complications and costs in cosmetic tourism also support evaluating access to follow‐up after travel, although those surgical populations differ from this service [40, 41].

From a service‐management perspective, the low return rate and substantial proportion of consultation‐only encounters suggest that new‐patient acquisition, counseling workload, and appointment capacity warrant separate measurement. The present billing data cannot estimate revenue, resource cost, treatment intensity, or the financial effect of any retention strategy.

### 4.8 Limitations

This study has four principal limitations. First, it was conducted at a single public tertiary academic center in Beijing. The case mix, fee structure, staffing, service capacity, referral environment, and patient‐origin composition may differ from those of private practices, commercial chains, multi‐center groups, or other regions. Changes in these institutional and external factors during 2015–2024 were not measured, so absolute utilization levels and temporal trends should not be treated as broadly generalizable.

Second, geographic origin was derived from a registration‐time residential address rather than a longitudinal measure of residence, travel burden, or accessibility. No patient had more than one distinct address in the available extract, and historical updating could not be verified; consequently, relocation, secondary residences, transportation mode, travel time, and socioeconomic context could not be assessed. Address information was unresolved for 13.7% of patients, although this was concentrated in the earliest years and affected only 0.40% of patient‐years in the 2022–2024 analytic window. Care obtained at other institutions was also invisible, so non‐return cannot be equated with discontinuation.

Third, treatment delivery and clinical outcomes were incompletely captured. Diagnosis‐derived categories were unvalidated indication proxies, were unavailable for all encounters, and could not represent combination treatment or the product, dose, or procedure actually administered. The hierarchical assignment of multi‐category patient‐years and the billing threshold used to identify treatment visits may have introduced misclassification; threshold sensitivity analyses showed broadly similar interval findings, but the direction of residual bias is uncertain. The administrative data also contained no patient‐reported outcomes, satisfaction, adverse events, or stated treatment preferences. Consecutive billed encounters may involve different modalities, and the ending diagnosis does not establish same‐product retreatment.

Finally, the models estimate associations rather than causal effects, and residual confounding is likely. Clustering accounts for repeated patient‐years but does not remove dependence of annual counts on time available after entry or unmeasured patient mix. First‐visit indication missingness varies over time, and selection into complete‐case strata may bias their estimates. Some Cox associations vary with follow‐up time; sparse late risk sets increase uncertainty. Indication, interaction, and threshold analyses remain exploratory, with nominal and selected BH‐adjusted *p* values reported. Confirmation requires multi‐center data containing procedure‐level and patient‐reported information.

Future studies should integrate procedure‐level treatment data with validated patient‐reported outcome measures, such as FACE‐Q, to determine whether different continuity patterns translate into differences in satisfaction, perceived age, safety, or treatment value [27, 42], and should collect travel time and patient‐stated intent so that preference and constraint can be distinguished.

## 5 Conclusion

In this 10‐year single‐center study, recorded service volume and repeat annual attendance increased; most patient‐years still comprised one visit. Adjusted annual count differences across administrative origin groups were small, while second‐visit and next‐year retention analyses suggested modest differences in observed re‐engagement. First‐visit indication strata showed exploratory heterogeneity, but these data do not identify travel burden, treatment‐specific maintenance, or the reasons for non‐return. The findings can guide prospective evaluation of follow‐up and communication strategies and require confirmation in other settings.

## Author Contributions

Ronghao Ouyang and Ximeng Jia conceived and designed the study, defined the patient‐origin classification and analytic framework, and led the methodology for the single‐center retrospective observational analysis. Ronghao Ouyang curated and cleaned the outpatient registration and billing data, performed the primary statistical analyses, including patient‐year construction and multivariable modeling, and drafted the manuscript. Ximeng Jia contributed to methodological refinement, results interpretation, and critical revision of the manuscript for important intellectual content. Ziming Zhang, Yutong Liang, and Benjie Li assisted with data verification, regional grouping checks, and interpretation of utilization and billing‐based findings. Mengzhe Qin prepared figures, tables, and visualizations of patient‐origin composition and utilization patterns, managed references, and supported manuscript editing. Jintian Hu provided senior supervision and clinical health‐services research guidance, reviewed the analyses, and approved the final version for submission. All authors reviewed and approved the manuscript.

## Funding

The authors have nothing to report.

## Ethics Statement

This retrospective study used routinely collected, de‐identified administrative data. The project was approved by the institutional ethics review board under approval No. 2026 (292). No study‐specific intervention or patient contact was undertaken, and no patient photographs or identifiable information are reported.

## Consent

A waiver of written informed consent was requested in the ethics submission. The supplied decision confirms project approval but does not explicitly state that the waiver was granted; written clarification from the reviewing committee is required before submission.

## Conflicts of Interest

The authors declare no conflicts of interest.

## Supporting information


**Appendix S1:** Supplementary tables and figures.
**Table S1:** Annual visit frequency and maintenance cadence by patient origin, six‐region classification (2022–2024).
**Table S2:** Diagnosis‐derived indication proxy distribution by patient origin, three‐level and six‐region classifications (2022–2024).
**Table S3:** Multivariable Poisson model for annual visit count, six‐region classification (2022–2024).
**Table S4:** Year‐over‐year retention by patient origin and index year (2015–2023).
**Table S5:** Completed STROBE checklist for cohort studies.
**Table S6:** Exploratory billing‐based annual total cost, cost per visit, and high‐cost patient‐years by patient origin under three thresholds (2022–2024).
**Table S7:** Exploratory clustered models for annual cost and high‐cost patient‐year under three thresholds, with same‐subset comparisons before and after indication adjustment (2022–2024).
**Table S8:** Age distribution by annual maintenance status and calendar year (2015–2024).
**Table S9:** Completeness of each extracted field, overall and by calendar year (2015–2024).
**Table S10:** Empirical low‐charge modes by calendar year and sensitivity of the treatment‐visit classification and observed consecutive treatment‐visit intervals to the billing threshold.
**Table S11:** Availability of registration address by calendar year, and comparison of characteristics between patients with and without a resolvable address.
**Table S12:** Annual visit count within indication proxy strata and patient‐clustered Wald test of the origin‐by‐indication interaction (2022–2024).
**Table S13:** Year‐over‐year retention by index‐year visit‐count strata, patient‐clustered interaction test, and same‐subset indication‐adjusted models (2015–2023 and 2022–2023).
**Table S14:** Survival diagnostics, horizon‐specific cumulative return estimates, and multiplicity checks.
**Figure S1:** Distribution of patient origins by province. Heatmap showing the number of unique patients by province in three periods (2015–2019, 2020–2022, and 2023–2024). Cell values indicate patient counts. Color intensity represents patient volume on a logarithmic scale. Provinces are grouped and labeled according to the six‐region classification.
**Figure S2:** Unique patients by Beijing district and period. A district‐level heatmap summarizes 2015–2019, 2020–2022, and 2023–2024. Counts are descriptive and not population‐standardized; a patient may appear in more than one period. Unresolved districts are shown separately. The institution's precise location is not displayed.
**Figure S3:** Annual visit‐count distribution by patient origin, violin plots (2022–2024). Violin plots showing the distribution of annual visit counts per patient‐year on a logarithmic y‐axis, for (A) the three‐level classification and (B) the six‐region classification. The width of each violin reflects the density of observations and the white marker indicates the median.
**Figure S4:** Exploratory billing‐based cost per visit by patient origin (2022–2024). Box plots showing the distribution of cost per visit (RMB) per patient‐year on a logarithmic y‐axis, for (A) the three‐level classification and (B) the six‐region classification. Boxes indicate the median and interquartile range (IQR), whiskers represent 1.5 × IQR, and points denote outliers. Cost per visit was calculated as annual total cost divided by annual visit count; patient‐years with any missing encounter charge or zero recorded total cost were excluded.
**Appendix S2:** Post‐extraction data derivation script.
**Appendix S3:** Statistical analysis and figure‐generation script.
**Appendix S4:** District‐level patient‐origin figure‐generation script.

## Data Availability

The data that support the findings of this study are derived from routinely collected clinical and administrative records at a single center and contain information that could compromise patient privacy. De‐identified data may be made available by the corresponding author upon reasonable request and subject to institutional approvals and applicable data‐use agreements.
