# Supplementary material for: Long‐Term Real‐World Utilization and Continuity Patterns in Injectable Aesthetic Dermatology: A 10‐Year Single‐Center Study
Source: J Cosmet Dermatol. 2026 Oct 14;25(10):e71234. doi: 10.1111/jocd.71234 (PMC13662613; doi:10.1111/jocd.71234)
Supplement: Supplementary file 1 [file JOCD-25-e71234-s001.zip › jocd71234-sup-0001-TableS1-S14-FigureS1-S4@Supplementary_Appendix_S1_Tables_and_Figures.docx]

**Supplementary Appendix S1. Supplementary tables and figures.**

Supplementary Table S1. Annual visit frequency and maintenance cadence by patient origin, six-region classification (2022-2024).

| **Region** | **N (patient-years)** | **Visits, median (IQR)** | **1 visit, No. (%)** | **2 visits, No. (%)** | **>=3 visits, No. (%)** | **>=2 visits (%)** |
| --- | --- | --- | --- | --- | --- | --- |
| Beijing | 13,775 | 1 (1-1) | 10,404 (75.5) | 2,253 (16.4) | 1,118 (8.1) | 24.5 |
| Jing-Jin-Ji | 1,507 | 1 (1-1) | 1,163 (77.2) | 218 (14.5) | 126 (8.4) | 22.8 |
| Other Eastern | 853 | 1 (1-1) | 642 (75.3) | 151 (17.7) | 60 (7.0) | 24.7 |
| Central | 683 | 1 (1-1) | 518 (75.8) | 122 (17.9) | 43 (6.3) | 24.2 |
| Western | 858 | 1 (1-1) | 678 (79.0) | 118 (13.8) | 62 (7.2) | 21.0 |
| Northeast | 551 | 1 (1-1) | 423 (76.8) | 81 (14.7) | 47 (8.5) | 23.2 |
| Overall P value |  | 0.265 (a) |  | 0.104 (b) |  |  |

(a) Kruskal-Wallis test across the six regions. (b) Pearson chi-square test for the 1/2/>=3 visit categories across the six regions. Jing-Jin-Ji denotes Tianjin and Hebei (excluding Beijing). Results are concordant with the three-level primary analysis presented in Table 3.

Supplementary Table S2. Diagnosis-derived indication proxy distribution by patient origin, three-level and six-region classifications (2022-2024).

Panel A. Three-level classification

| **Patient origin** | **Neuromodulator-type** | **Volume/contour-type** | **Skin-quality-type** | **Post-procedure review** | **Other/unclassified** | **Total classifiable** |
| --- | --- | --- | --- | --- | --- | --- |
| Beijing | 5,358 (45.6) | 3,012 (25.6) | 2,794 (23.8) | 471 (4.0) | 118 (1.0) | 11,753 |
| Nearby nonlocal | 531 (40.1) | 400 (30.2) | 231 (17.4) | 136 (10.3) | 27 (2.0) | 1,325 |
| Distant nonlocal | 1,094 (42.3) | 811 (31.3) | 405 (15.7) | 243 (9.4) | 34 (1.3) | 2,587 |

Values are No. (%) of classifiable patient-years. Pearson chi-square = 307.79, df = 8, P <0.001.

Panel B. Six-region classification

| **Patient origin** | **Neuromodulator-type** | **Volume/contour-type** | **Skin-quality-type** | **Post-procedure review** | **Other/unclassified** | **Total classifiable** |
| --- | --- | --- | --- | --- | --- | --- |
| Beijing | 5,358 (45.6) | 3,012 (25.6) | 2,794 (23.8) | 471 (4.0) | 118 (1.0) | 11,753 |
| Jing-Jin-Ji | 531 (40.1) | 400 (30.2) | 231 (17.4) | 136 (10.3) | 27 (2.0) | 1,325 |
| Other Eastern | 318 (42.3) | 263 (35.0) | 92 (12.2) | 69 (9.2) | 10 (1.3) | 752 |
| Central | 257 (42.8) | 178 (29.6) | 97 (16.1) | 60 (10.0) | 9 (1.5) | 601 |
| Western | 341 (44.8) | 215 (28.2) | 141 (18.5) | 62 (8.1) | 3 (0.4) | 762 |
| Northeast | 178 (37.7) | 155 (32.8) | 75 (15.9) | 52 (11.0) | 12 (2.5) | 472 |

Pearson chi-square = 342.51, df = 20, P <0.001. Categories were derived from the free-text primary outpatient diagnosis using the hierarchical rule described in the Methods and are indication proxies, not procedure or product categories. Of 18,227 patient-years, 15,665 (85.9%) could be classified.

Supplementary Table S3. Multivariable Poisson model for annual visit count, six-region classification (2022-2024).

| **Origin (vs Beijing)** | **Adjusted RR (95% CI)** | **P** |
| --- | --- | --- |
| Jing-Jin-Ji | 0.994 (0.953–1.036) | 0.773 |
| Other Eastern | 0.964 (0.924–1.005) | 0.088 |
| Central | 0.952 (0.911–0.995) | 0.030 |
| Western | 0.945 (0.898–0.995) | 0.030 |
| Northeast | 0.969 (0.912–1.030) | 0.307 |

Poisson regression with standard errors clustered by patient, adjusted for calendar year, age and sex. Unit of analysis: patient-year. N = 18,227. Reference: Beijing. The three-level primary model is presented in Table 4.

Supplementary Table S4. Year-over-year retention by patient origin and index year (2015-2023).

| **Patient origin** | **2015** | **2016** | **2017** | **2018** | **2019** | **2020** | **2021** | **2022** | **2023** | **Pooled 2015-2023** | **Index years 2022-2023** |
| --- | --- | --- | --- | --- | --- | --- | --- | --- | --- | --- | --- |
| Beijing | 35.4 | 40.4 | 28.8 | 27.7 | 20.7 | 27.0 | 18.0 | 25.7 | 26.2 | 25.5 | 26.0 |
| Nearby nonlocal | 45.2 | 40.2 | 24.5 | 15.5 | 10.4 | 18.8 | 15.7 | 21.6 | 22.0 | 20.4 | 21.9 |
| Distant nonlocal | 30.7 | 36.0 | 19.1 | 16.7 | 8.0 | 13.8 | 9.7 | 19.9 | 21.5 | 17.9 | 21.2 |

Values are percentages returning in the following calendar year among patient-years observed in the index year. Pooled and recent crude chi-square comparisons are descriptive because patient-years repeat within patients; clustered adjusted analyses are in Supplementary Table S13. This measure reduces the cumulative-history dependence of returning-patient-year status but remains affected by patient composition.

Supplementary Table S5 is the completed STROBE checklist, reproduced below.

Supplementary Table S5. Completed STROBE checklist for cohort studies.

STROBE Statement: checklist of items that should be included in reports of observational studies (cohort design). Item numbering follows von Elm et al., J Clin Epidemiol 2008;61:344-349. Locations refer to the second revised manuscript (R2). Items whose content changed at this revision are marked with a dagger.

| **Item No.** | **Recommendation** | **Reported** | **Location in revised manuscript** |
| --- | --- | --- | --- |
| 1a | Indicate the study's design with a commonly used term in the title or the abstract | Yes | Title ("A 10-Year Single-Center Study"); Abstract, Methods ("retrospectively analyzed outpatient registration and billing records") |
| 1b | Provide in the abstract an informative and balanced summary of what was done and what was found | Yes | Abstract (structured: Background, Aims, Methods, Results, Conclusion; 253 words). The Abstract now also reports that most patient-years in every origin group comprised a single visit and describes the geographic association as modest |
| 2 | Explain the scientific background and rationale for the investigation being reported | Yes | Introduction, paragraphs 1-4 (shortened at this revision; pharmacologic benchmark detail retained in Discussion, where the comparison is made) |
| 3 | State specific objectives, including any prespecified hypotheses | Yes | Introduction, final paragraph |
| 4 | Present key elements of study design early in the paper | Yes | Methods, "Study design and setting" |
| 5 † | Describe the setting, locations, and relevant dates, including periods of recruitment, exposure, follow-up, and data collection | Yes | Methods, "Study design and setting": public, hospital-based tertiary academic plastic surgery centre in Beijing, China; January 1, 2015 to December 31, 2024; patients attend predominantly on their own initiative rather than by inter-institutional referral; definition of the COVID-19 pandemic period (2020-2022) and post-pandemic period (2023-2024). Institutional changes during the period (fee schedule, staffing, online outreach) are described in Discussion, "Service-level changes during the study period" |
| 6a † | Give the eligibility criteria, and the sources and methods of selection of participants. Describe methods of follow-up | Yes | Methods: study design, data extraction, and outcomes. All recorded encounters in 2015–2024 were included; origin comparisons exclude unresolvable origin. No additional study-driven recruitment or active follow-up occurred. |
| 6b | For matched studies, give matching criteria and number of exposed and unexposed | Not applicable | No matching was performed |
| 7 † | Clearly define all outcomes, exposures, predictors, potential confounders, and effect modifiers. Give diagnostic criteria, if applicable | Yes | Methods, "Terminology" (explicit definitions of maintenance-based model of care, cadence, continuity of care, patient-origin composition, indication proxy, and treatment visit); "Outcomes"; "Patient-origin classification and periodization" (exposure); "Diagnosis-derived indication proxy categories"; "Treatment visits versus consultation-only visits"; confounders (calendar year, age, sex, index-year visit count) and the effect modifier examined (indication proxy) specified in "Statistical analysis" |
| 8 † | For each variable of interest, give sources of data and details of methods of assessment. Describe comparability of assessment methods if there is more than one group | Yes | Methods: data source; indication proxies; origin classification; billing definition. Supplementary Appendices S2 and S3 provide post-extraction derivation and aggregate analysis scripts, not the hospital query. Products and clinical outcomes were unavailable; proxies were not validated against a treatment register. |
| 9 † | Describe any efforts to address potential sources of bias | Yes | Methods and Limitations: address and diagnosis missingness, within-patient province reconciliation, diagnosis hierarchy, selection into strict first-visit strata, incomplete charges, imperfect proportional hazards, and unobserved care elsewhere. |
| 10 | Explain how the study size was arrived at | Yes | Methods, "Study design and setting" and Results, first paragraph: the study included all eligible encounters in the 10-year period; no a priori sample-size calculation was performed as the analysis was a complete-census retrospective review |
| 11 † | Explain how quantitative variables were handled in the analyses. If applicable, describe which groupings were chosen and why | Yes | Methods, "Data source and data extraction" (patient-year construction; annual visit count; maintenance categories 1, 2, >=3 visits); "Patient-origin classification and periodization" (three-level primary and six-region sensitivity grouping, with rationale); "Treatment visits versus consultation-only visits" (empirical derivation of the fee threshold); "Outcomes" (high-cost thresholds: >=10,000 RMB, top quartile, top decile); "Statistical analysis" (log transformation of annual cost; calendar year continuous in the trend model and categorical in regional models; index-year visit count categorized as 1, 2, >=3) |
| 12a | Describe all statistical methods, including those used to control for confounding | Yes | Methods, Statistical analysis. Calendar year categorical in patient-year models except the continuous temporal-trend term; Cox baseline hazards stratified by first-visit year; patient-clustered covariance for patient-year models; categorical visit-count strata; Cox survival models and time-specific sensitivity estimates. |
| 12b † | Describe any methods used to examine subgroups and interactions | Yes | Table 6: Cox origin-by-indication likelihood-ratio interaction; S12: clustered Poisson origin-by-indication Wald test; S13: clustered logistic origin-by-visit-count Wald test. S14 provides selected BH adjustments and proportional-hazards checks. |
| 12c † | Explain how missing data were addressed | Yes | S9 reports source-field completeness. Unresolvable origins remain excluded; same-patient province propagation is explicitly reported. First diagnoses are never backfilled. Cost analyses exclude any missing charge and zero-cost patient-years; indication models use complete subsets with same-subset base comparisons. |
| 12d | If applicable, explain how loss to follow-up was addressed | Yes | Time to second visit is administratively censored at the end of December 31, 2024. Care elsewhere and reasons for non-return are unknown. S14 examines time dependence; the follow-up sensitivity requires first attendance before July 2023. |
| 12e † | Describe any sensitivity analyses | Yes | Methods, "Statistical analysis", final paragraph. Sensitivity analyses comprise: the six-region geographic grouping (Supplementary Tables S1, S3); the year-specific modal registration fee and fixed thresholds of 50, 100 and 200 RMB for the treatment-visit definition (Supplementary Table S10); winsorization of annual cost; alternative high-cost thresholds at the top quartile and top decile (Supplementary Tables S6, S7); exclusion of consecutive treatment-visit intervals shorter than 30 days; restriction to patients with >=18 months of potential follow-up; cost and high-cost models refitted with additional adjustment for indication proxy (Supplementary Table S7); retention models refitted with additional adjustment for indication proxy and reported for two index-year windows (Supplementary Table S13); and time-to-event and cadence analyses stratified by indication proxy (Table 6, Supplementary Table S12) |
| 13a † | Report numbers of individuals at each stage of the study | Yes | Results: 48,798 encounters, 29,437 patients; 25,399 resolvable and 4,038 unresolvable origins. Recent patient-year cohort n = 18,227. Survival n = 25,399; strict four-category first-visit strata n = 17,008; cost cohort n = 18,181. Model-specific n appears in tables. |
| 13b † | Give reasons for non-participation at each stage | Yes | Methods and Results state branch-specific exclusions: origin unresolvable; first-visit diagnosis missing or outside four categories; covariate missingness; any missing encounter charge or zero annual cost. The final study day is fully included. |
| 13c | Consider use of a flow diagram | Not included | Participant counts and reasons for exclusions are reported numerically by analysis branch; no flow diagram is supplied. |
| 14a † | Give characteristics of study participants and information on exposures and potential confounders | Yes | Table 1 (age, sex, visit volume by year); Table 2 (patient-origin composition); Supplementary Table S8 (age by maintenance status); Supplementary Table S11 (characteristics of patients with and without a resolvable address) |
| 14b † | Indicate the number of participants with missing data for each variable of interest | Yes | S9: field-level completeness. Results and Table 6: first-visit diagnosis available for 17,184/25,399. S11 displays missing first diagnoses explicitly. Cost exclusions are quantified in Results and S6. |
| 14c | Summarise follow-up time | Yes | Results, Time to second visit: distribution of observed time to event/censoring and potential observation to study end; S14 provides horizon-specific probabilities with CIs and figures give risk counts. |
| 15 † | Report numbers of outcome events or summary measures over time | Yes | Tables 1, 3, 5, and 6; 7,565 overall second visits and 5,418 in strict first-visit strata. Retention events are reported in S13a. |
| 16a † | Give unadjusted estimates and, if applicable, confounder-adjusted estimates and their precision. Make clear which confounders were adjusted for and why they were included | Yes | Crude distributions and absolute return probabilities accompany adjusted models. Patient-year CIs are clustered by patient. Adjustment variables and fixed model structure are described in Methods. Paired same-subset cost and retention models separate selection from indication adjustment. |
| 16b | Report category boundaries when continuous variables were categorized | Yes | Methods, "Data source and data extraction" (1, 2, >=3 visits); "Treatment visits versus consultation-only visits" (>60 RMB primary; year-specific and alternative thresholds); "Outcomes" (high-cost thresholds, with the corresponding RMB values reported in Results); "Patient-origin classification and periodization" (period boundaries); "Statistical analysis" (index-year visit count 1, 2, >=3) |
| 16c | If relevant, consider translating estimates of relative risk into absolute risk for a meaningful time period | Yes | Tables 3 and 5 provide absolute attendance distributions and horizon-specific cumulative return alongside relative effects. No intervention effect, clinical benefit, or number needed to treat is inferred from these observational associations. |
| 17 † | Report other analyses done - e.g. analyses of subgroups and interactions, and sensitivity analyses | Yes | Results, "Diagnosis-derived indication proxy mix"; "Time to second visit stratified by indication proxy"; "Treatment visits and observed billed-encounter intervals"; "Exploratory billing-based outcomes"; Table 6; Supplementary Tables S1-S4, S6-S14; Supplementary Figures S3, S4; Figure 6 |
| 18 | Summarise key results with reference to study objectives | Yes | Discussion, first paragraph; Conclusion |
| 19 † | Discuss limitations of the study, taking into account sources of potential bias or imprecision. Discuss both direction and magnitude of any potential bias | Yes | Discussion, Limitations: four paragraphs on single-center design; origin and unobserved external care; incomplete measurement of treatment; residual confounding, repeated patient-years, time dependence, and exploratory comparisons. |
| 20 † | Give a cautious overall interpretation of results considering objectives, limitations, multiplicity of analyses, results from similar studies, and other relevant evidence | Yes | Discussion and Conclusion avoid interpreting origin as distance, billing as treatment intensity, or proxy-stratum differences as mechanisms. S14 reports selected multiplicity checks. Clinical reminder timing and reasons for attendance remain unmeasured. |
| 21 † | Discuss the generalisability (external validity) of the study results | Yes | Discussion, Interpreting the setting: both direction and magnitude of findings require external validation in public and private settings; the estimates are not proposed as reference standards. |
| 22 | Give the source of funding and the role of the funders for the present study | Yes | Funding statement (title page) |

† Content of this item changed at the second revision.

Ethics approval information is provided on the separate title page.

Supplementary Table S6. Exploratory billing-based annual total cost, cost per visit, and high-cost patient-years by patient origin under three thresholds (2022-2024).

| **Origin** | **Patient-years, n** | **Annual cost, RMB, median (IQR)** | **Cost/visit, RMB, median (IQR)** | **≥10,000 RMB, n (%)** | **Top quartile, n (%)** | **Top decile, n (%)** |
| --- | --- | --- | --- | --- | --- | --- |
| Beijing | 13,734 | 5,142 (60–11,279) | 4,843 (60–8,269) | 4,092 (29.8) | 3,293 (24.0) | 1,278 (9.3) |
| Nearby nonlocal | 1,506 | 5,050 (60–12,550) | 4,629 (60–9,511) | 489 (32.5) | 398 (26.4) | 170 (11.3) |
| Distant nonlocal | 2,941 | 5,530 (60–13,102) | 5,035 (60–10,054) | 1,012 (34.4) | 855 (29.1) | 371 (12.6) |
| Crude P |  | 0.003 | <0.001 | <0.001 | <0.001 | <0.001 |

Abbreviations: IQR, interquartile range; RMB, renminbi. Excluded: 38 patient-years with any missing encounter charge and 8 with complete zero total cost. Quantile thresholds were derived once from the 18,181 eligible patient-years: 11,557 RMB (75th percentile) and 19,401.5 RMB (90th percentile). Crude Kruskal–Wallis and chi-square P values are descriptive; clustered adjusted models are in S7. Charges do not establish clinical treatment intensity.

**Supplementary Table S7. Exploratory clustered models for annual cost and high-cost patient-year under three thresholds, with same-subset comparisons before and after indication adjustment (2022–2024).**

| **Outcome** | **Model** | **Patient-years, n** | **Nearby effect (95% CI)** | **P** | **Distant effect (95% CI)** | **P** |
| --- | --- | --- | --- | --- | --- | --- |
| Annual cost ratio | Base, all eligible | 18,181 | 0.92 (0.81–1.05) | 0.224 | 0.96 (0.87–1.06) | 0.394 |
|  | Base, same subset | 15,457 | 0.99 (0.87–1.11) | 0.813 | 1.01 (0.92–1.11) | 0.847 |
|  | + indication proxy | 15,457 | 1.14 (1.01–1.28) | 0.031 | 1.15 (1.05–1.25) | 0.003 |
| ≥10,000 RMB, aOR | Base, all eligible | 18,181 | 1.09 (0.97–1.23) | 0.160 | 1.14 (1.04–1.25) | 0.004 |
|  | Base, same subset | 15,457 | 1.13 (1.00–1.28) | 0.048 | 1.18 (1.07–1.29) | <0.001 |
|  | + indication proxy | 15,457 | 1.27 (1.12–1.44) | <0.001 | 1.29 (1.17–1.42) | <0.001 |
| Top quartile (11,557 RMB), aOR | Base, all eligible | 18,181 | 1.08 (0.95–1.23) | 0.237 | 1.19 (1.08–1.30) | <0.001 |
|  | Base, same subset | 15,457 | 1.11 (0.98–1.27) | 0.106 | 1.22 (1.10–1.34) | <0.001 |
|  | + indication proxy | 15,457 | 1.24 (1.08–1.41) | 0.002 | 1.32 (1.19–1.46) | <0.001 |
| Top decile (19,401.5 RMB), aOR | Base, all eligible | 18,181 | 1.18 (0.99–1.40) | 0.071 | 1.28 (1.12–1.46) | <0.001 |
|  | Base, same subset | 15,457 | 1.20 (1.01–1.44) | 0.043 | 1.30 (1.14–1.48) | <0.001 |
|  | + indication proxy | 15,457 | 1.32 (1.10–1.58) | 0.002 | 1.39 (1.21–1.58) | <0.001 |

Indication proxy mix by origin group, % of patient-years:

| **Origin** | **Neuromodulator-type** | **Volume/contour-type** | **Skin-quality-type** | **Post-procedure review** | **Other/unclassified** |
| --- | --- | --- | --- | --- | --- |
| Beijing | 45.6 | 25.6 | 23.8 | 4.0 | 1.0 |
| Nearby nonlocal | 40.1 | 30.1 | 17.4 | 10.3 | 2.0 |
| Distant nonlocal | 42.3 | 31.3 | 15.7 | 9.4 | 1.3 |

High-cost (>=10,000 RMB) percentage by origin and indication proxy:

| **Indication proxy** | **Beijing** | **Nearby nonlocal** | **Distant nonlocal** |
| --- | --- | --- | --- |
| Neuromodulator-type | 36.0 | 40.1 | 43.2 |
| Volume/contour-type | 38.1 | 42.9 | 44.2 |
| Skin-quality-type | 35.0 | 42.9 | 40.7 |
| Post-procedure review | 2.8 | 4.4 | 5.8 |

All models adjust for categorical calendar year, age, and sex, with patient-clustered standard errors and Beijing as reference. Annual-cost effects are exponentiated log-linear coefficients; high-cost effects are odds ratios. The same-subset base and indication-adjusted models use identical four-category complete cases; all-eligible base models are shown separately. Quantile thresholds are fixed from the full eligible cost sample. The indication mix panel includes Other/unclassified so that percentages sum to approximately 100%. Neither adjustment nor the descriptive mix establishes a mechanism. Product, dose, package billing, physician factors, and clinical outcomes were unavailable.

Supplementary Table S8. Age distribution by annual maintenance status and calendar year (2015-2024).

| **Year** | **1 visit, n** | **1 visit, age median (IQR)** | **>=2 visits, n** | **>=2 visits, age median (IQR)** |
| --- | --- | --- | --- | --- |
| 2015 | 2,184 | 31.1 (25.8-39.2) | 466 | 31.0 (26.3-39.5) |
| 2016 | 2,214 | 32.1 (26.4-40.6) | 427 | 31.5 (26.7-40.3) |
| 2017 | 1,988 | 32.6 (27.0-41.6) | 397 | 32.2 (27.1-39.3) |
| 2018 | 1,947 | 32.2 (26.5-40.0) | 344 | 33.2 (28.2-40.6) |
| 2019 | 2,173 | 32.3 (26.9-40.0) | 433 | 33.9 (28.4-41.7) |
| 2020 | 2,073 | 31.9 (27.1-38.3) | 386 | 33.5 (27.3-39.7) |
| 2021 | 3,076 | 32.9 (27.8-39.0) | 761 | 33.6 (28.1-40.1) |
| 2022 | 2,225 | 34.5 (29.3-40.4) | 584 | 36.5 (30.8-42.8) |
| 2023 | 4,852 | 34.9 (29.5-41.5) | 1,290 | 36.4 (30.7-43.3) |
| 2024 | 6,806 | 35.6 (29.8-41.8) | 2,543 | 37.2 (31.5-43.5) |

Age was computed at the median visit date within each patient-year. Patients attending at least twice within a calendar year were slightly older than those attending once; the calendar-year trend in maintenance utilization persisted after adjustment for age and sex (see Results).

**Supplementary Table S9. Completeness of each extracted field, overall and by calendar year (2015-2024).**

| **Extracted field** | **Overall** | **2015** | **2016** | **2017** | **2018** | **2019** | **2020** | **2021** | **2022** | **2023** | **2024** |
| --- | --- | --- | --- | --- | --- | --- | --- | --- | --- | --- | --- |
| De-identified patient identifier | 100.0 | 100.0 | 100.0 | 100.0 | 100.0 | 100.0 | 100.0 | 100.0 | 100.0 | 100.0 | 100.0 |
| Encounter serial number | 100.0 | 100.0 | 100.0 | 100.0 | 100.0 | 100.0 | 100.0 | 100.0 | 100.0 | 100.0 | 100.0 |
| De-identified medical record number | 83.5 | 83.6 | 80.3 | 82.5 | 82.2 | 82.2 | 84.0 | 81.3 | 84.4 | 83.4 | 85.5 |
| Sex | 100.0 | 100.0 | 100.0 | 100.0 | 100.0 | 100.0 | 100.0 | 100.0 | 100.0 | 100.0 | 100.0 |
| Date of birth | 100.0 | 100.0 | 100.0 | 100.0 | 100.0 | 99.6 | 99.9 | 100.0 | 100.0 | 100.0 | 100.0 |
| Encounter date | 100.0 | 100.0 | 100.0 | 100.0 | 100.0 | 100.0 | 100.0 | 100.0 | 100.0 | 100.0 | 100.0 |
| Primary outpatient diagnosis (free text) | 70.1 | 54.1 | 49.8 | 56.7 | 57.3 | 57.8 | 59.5 | 53.8 | 60.2 | 81.2 | 91.5 |
| Residential address recorded at registration | 89.4 | 38.4 | 45.4 | 72.1 | 89.1 | 95.3 | 99.3 | 99.8 | 99.6 | 99.6 | 99.5 |
| Total visit-level charge | 99.9 | 100.0 | 100.0 | 100.0 | 100.0 | 100.0 | 99.7 | 99.7 | 99.7 | 99.9 | 99.9 |
| *Derived:* resolvable province | 89.4 | 38.4 | 45.4 | 72.0 | 89.0 | 95.1 | 99.3 | 99.8 | 99.6 | 99.6 | 99.5 |

Values are the percentage of encounters with a non-missing value. N = 48,798 encounters. Age was derived from date of birth and encounter date. The province field is derived from the free-text address by the parser given in Supplementary Appendix S2; encounters whose address could not be resolved to a province are counted as missing here and labelled Unknown in analysis. Missing charges (65 encounters) are unclassified for billing proxies. A province can be reconciled from another record belonging to the same patient; this is explicitly distinguished from diagnosis imputation in Methods.

**Supplementary Table S10. Empirical low-charge modes by calendar year and sensitivity of the treatment-visit classification and observed consecutive treatment-visit intervals to the billing threshold.**

***S10a. Empirical annual low-charge mode***

| **Year** | **Encounters** | **Modal low charge (RMB)** | **n at modal value** | **% of encounters <=60 RMB** | **% <=modal fee** |
| --- | --- | --- | --- | --- | --- |
| 2015 | 3,259 | 28 | 593 | 34.6 | 34.2 |
| 2016 | 3,216 | 14 | 620 | 39.0 | 19.9 |
| 2017 | 2,905 | 60 | 298 | 31.4 | 31.4 |
| 2018 | 2,740 | 60 | 456 | 32.1 | 32.1 |
| 2019 | 3,200 | 50 | 582 | 32.8 | 18.9 |
| 2020 | 2,996 | 50 | 637 | 35.2 | 21.8 |
| 2021 | 5,004 | 50 | 1,434 | 42.7 | 30.0 |
| 2022 | 3,707 | 50 | 780 | 38.0 | 21.3 |
| 2023 | 8,071 | 50 | 1,706 | 29.6 | 21.6 |
| 2024 | 13,700 | 60 | 3,259 | 31.1 | 31.1 |

The low-charge mode was identified empirically within 1–100 RMB in each year. These values are compatible with registration/consultation charges but were not checked against an audited fee schedule. No inflation adjustment was applied. The primary threshold is 60 RMB; lower or higher thresholds may misclassify both low-cost procedures and nonprocedural charges.

***S10b. Treatment-visit percentage by year under each threshold***

| **Year** | **Year-specific modal fee** | **50 RMB** | **60 RMB (primary)** | **100 RMB** | **200 RMB** |
| --- | --- | --- | --- | --- | --- |
| 2015 | 65.8 | 65.5 | 65.4 | 65.3 | 65.1 |
| 2016 | 80.1 | 61.2 | 61.0 | 60.9 | 60.3 |
| 2017 | 68.6 | 79.1 | 68.6 | 68.2 | 67.5 |
| 2018 | 67.9 | 84.7 | 67.9 | 67.8 | 66.9 |
| 2019 | 81.1 | 81.1 | 67.2 | 66.8 | 66.2 |
| 2020 | 77.9 | 77.9 | 64.5 | 64.5 | 63.4 |
| 2021 | 69.7 | 69.7 | 57.0 | 56.8 | 56.4 |
| 2022 | 78.4 | 78.4 | 61.7 | 61.4 | 58.5 |
| 2023 | 78.3 | 78.3 | 70.3 | 70.0 | 67.9 |
| 2024 | 68.7 | 92.5 | 68.7 | 68.3 | 66.7 |
| **All years** | **73.0** | **80.0** | **66.1** | **65.8** | **64.5** |

The ranking of years is threshold-dependent: the 2021 minimum is present under the fixed 60, 100 and 200 RMB thresholds but not under the year-specific fee, where 2015 is lowest, nor at 50 RMB, where 2016 is lowest. The year-to-year series should therefore not be interpreted as a trend in practice.

***S10c.*** Po***s***iti***ve interval***s ***between consecutive*** billi***n***g-po***sit***ive encounter***s, by threshold***

| **Threshold** | **Billed encounters, %** | **Positive intervals, n** | **Median days, all** | **Neuromodulator** | **Volume/contour** | **Skin-quality** | **Neuro 90–<180 d, %** | **Neuro ≥180 d, %** |
| --- | --- | --- | --- | --- | --- | --- | --- | --- |
| Year-specific modal fee | 73.0 | 14,576 | 176 | 219 | 88 | 185 | 20.9 | 65.1 |
| 50 RMB | 80.0 | 15,774 | 165 | 210 | 78 | 182 | 20.4 | 61.2 |
| 60 RMB (primary) | 66.1 | 13,240 | 187 | 224 | 105 | 194 | 21.1 | 67.4 |
| 100 RMB | 65.8 | 13,152 | 188 | 224 | 105 | 195 | 21.0 | 67.4 |
| 200 RMB | 64.5 | 12,847 | 190 | 224 | 98 | 196 | 21.1 | 67.7 |

Only integer-day intervals >0 are included consistently. Diagnosis category is assigned from the ending encounter; the preceding encounter may carry a different or missing diagnosis. The 3–6-month comparison is 90–<180 days and the ≥6-month comparison is ≥180 days, so exactly 180 days is included. Missing charges remain unclassified; billed-encounter percentages use all encounters as denominator. These are not validated same-product retreatment intervals.

**Supplementary Table S11. Availability of registration address by calendar year, and comparison of characteristics between patients with and without a resolvable address.**

***S11a. Patient-years with an unresolvable origin, by calendar year***

| **Year** | **Patient-years, n** | **Unresolvable origin, n** | **Unresolvable, %** |
| --- | --- | --- | --- |
| 2015 | 2,650 | 1,732 | 65.36 |
| 2016 | 2,641 | 1,560 | 59.07 |
| 2017 | 2,386 | 744 | 31.18 |
| 2018 | 2,292 | 265 | 11.56 |
| 2019 | 2,618 | 131 | 5.00 |
| 2020 | 2,461 | 18 | 0.73 |
| 2021 | 3,837 | 8 | 0.21 |
| 2022 | 2,810 | 13 | 0.46 |
| 2023 | 6,142 | 18 | 0.29 |
| 2024 | 9,349 | 43 | 0.46 |
| 2015-2019 | 12,587 | 4,432 | 35.21 |
| 2020-2022 | 9,108 | 39 | 0.43 |
| **2022-2024 (analytic window)** | 18,301 | 74 | 0.40 |
| **All years** | 37,186 | 4,532 | 12.19 |

***S11b. Characteristics of patients with and without a resolvable address***

| **Characteristic** | **Resolvable (n = 25,399)** | **Unresolvable (n = 4,038)** | **P** |
| --- | --- | --- | --- |
| Age at first visit, years, median (IQR) | 33.7 (28.1–40.4) | 31.9 (26.1–41.1) | <0.001 |
| Female, % | 91.5 | 92.4 | 0.066 |
| Observed visits, median (IQR) | 1 (1–2) | 1 (1–1) | <0.001 |
| Second visit, % | 29.8 | 17.7 | <0.001 |
| Billed encounters >60 RMB, median (IQR) | 1 (0–1) | 1 (0–1) | <0.001 |
| Complete recorded total charge, RMB, median (IQR) | 5,035 (60–11,912) | 2,767 (28–6,698) | <0.001 |
| First visit in 2015–2017, % | 11.1 | 91.9 | <0.001 |
| First visit in 2022–2024, % | 56.6 | 1.6 |  |
| First-visit proxy: Neuromodulator-type | 27.7 | 21.2 | <0.001 |
| First-visit proxy: Volume/contour-type | 13.5 | 0.6 |  |
| First-visit proxy: Skin-quality-type | 22.4 | 19.0 |  |
| First-visit proxy: Post-procedure review | 3.4 | 0.1 |  |
| First-visit proxy: Other/unclassified | 0.7 | 0.2 |  |
| First-visit proxy: Missing diagnosis | 32.3 | 58.9 |  |

First-visit diagnosis is taken from the first encounter row without skipping missing values. Indication percentages use all patients in each address group, with Other/unclassified and Missing diagnosis displayed explicitly. Recorded total-charge summaries exclude patients with any missing charge (eligible n = 25,338 and 4,034). Missing origin is associated with era and observed characteristics; its cause and direction of selection bias cannot be established. Continuous-variable P values use Mann–Whitney tests and categorical P values chi-square tests. The first-visit diagnosis P value is for the overall six-category distribution.

**Supplementary Table S12. Annual visit count within indication proxy strata and patient-clustered Wald test of the origin-by-indication interaction (2022–2024).**

***S12a. Descriptive***

| **Indication proxy** | **Beijing: >=2 visits, % (n)** | **Nearby nonlocal: >=2 visits, % (n)** | **Distant nonlocal: >=2 visits, % (n)** | **P** |
| --- | --- | --- | --- | --- |
| Neuromodulator-type | 30.1 (5,358) | 25.8 (531) | 28.1 (1,094) | 0.065 |
| Volume/contour-type | 27.4 (3,012) | 24.5 (400) | 21.2 (811) | 0.001 |
| Skin-quality-type | 24.5 (2,794) | 18.6 (231) | 20.0 (405) | 0.026 |
| Post-procedure review | 26.5 (471) | 40.4 (136) | 36.6 (243) | 0.001 |

***S12b. Poisson models for annual visit count within indication strata***

| **Indication proxy** | **Patient-years, n** | **Nearby aRR (95% CI)** | **P** | **Distant aRR (95% CI)** | **P** |
| --- | --- | --- | --- | --- | --- |
| Neuromodulator-type | 6,983 | 0.935 (0.882–0.992) | 0.025 | 0.937 (0.900–0.976) | 0.002 |
| Volume/contour-type | 4,223 | 0.994 (0.917–1.077) | 0.883 | 0.927 (0.877–0.979) | 0.006 |
| Skin-quality-type | 3,430 | 0.937 (0.864–1.017) | 0.118 | 0.940 (0.886–0.998) | 0.043 |
| Post-procedure review | 850 | 1.365 (1.131–1.647) | 0.001 | 1.095 (0.974–1.232) | 0.130 |

Poisson models adjust for categorical calendar year, age, and sex, with patient-clustered standard errors and Beijing as reference. The joint origin-by-indication Wald test has χ² = 17.31, df = 6, P = 0.008; BH-adjusted P = 0.012 across three principal interaction tests. Strata use the dominant recorded patient-year indication under the stated hierarchy, not a confirmed administered treatment. Crude descriptive P values in panel A do not account for repeated patient-years; subgroup multiplicity checks are in S14.

**Supplementary Table S13. Year-over-year retention by index-year visit-count strata, patient-clustered interaction test, and same-subset indication-adjusted models (2015–2023 and 2022–2023).**

***S13a. Unadjusted retention, index years 2015-2023***

| **Index-year visits** | **Beijing, % (n/N)** | **Nearby nonlocal, % (n/N)** | **Distant nonlocal, % (n/N)** | **P** |
| --- | --- | --- | --- | --- |
| 1 visit | 19.3 (2,736/14,194) | 17.1 (238/1,393) | 13.9 (444/3,185) | <0.001 |
| 2 visits | 45.0 (1,198/2,663) | 33.2 (71/214) | 33.5 (153/457) | <0.001 |
| >=3 visits | 61.2 (613/1,002) | 44.2 (34/77) | 50.9 (83/163) | 0.001 |
| Crude association across visit-count categori**es** | <0.001 | <0.001 | <0.001 |  |

***S13b. Origin effect estimated separately within each engagement stratum***

| **Index-year visits** | **Patient-years, n** | **Nearby aOR (95% CI)** | **P** | **Distant aOR (95% CI)** | **P** |
| --- | --- | --- | --- | --- | --- |
| 1 visit | 18,772 | 0.84 (0.71–1.00) | 0.053 | 0.66 (0.57–0.75) | <0.001 |
| 2 visits | 3,334 | 0.59 (0.44–0.80) | <0.001 | 0.56 (0.45–0.71) | <0.001 |
| >=3 visits | 1,242 | 0.55 (0.35–0.86) | 0.009 | 0.64 (0.44–0.92) | 0.017 |

All models adjust for categorical index year, age, and sex and use standard errors clustered by patient. Joint origin-by-count Wald test: χ² = 9.00, df = 4, P = 0.061; BH-adjusted P = 0.061. In the main-effects model, ORs relative to one index-year visit are 3.27 (95% CI 3.02–3.55) for two visits and 6.34 (95% CI 5.60–7.17) for three or more. A nonsignificant interaction is not evidence of equivalence across strata.

***S13c. Retention models by index-year window, with and without adjustment for indication proxy***

| **Model** | **Index years** | **Patient-years, n** | **Nearby aOR (95% CI)** | **Distant aOR (95% CI)** |
| --- | --- | --- | --- | --- |
| Base, all eligible | 2015–2023 | 23,348 | 0.75 (0.64–0.87) | 0.63 (0.56–0.71) |
| Base, same subset | 2015–2023 | 15,734 | 0.73 (0.62–0.87) | 0.66 (0.58–0.75) |
| + indication proxy | 2015–2023 | 15,734 | 0.77 (0.65–0.90) | 0.68 (0.60–0.78) |
| Base, all eligible | 2022–2023 | 8,921 | 0.82 (0.65–1.04) | 0.76 (0.65–0.89) |
| Base, same subset | 2022–2023 | 6,838 | 0.79 (0.62–1.02) | 0.75 (0.63–0.89) |
| + indication proxy | 2022–2023 | 6,838 | 0.86 (0.67–1.10) | 0.78 (0.66–0.93) |

Logistic models adjust for categorical index year, age, sex, and continuous index-year visit count, with patient-clustered standard errors and Beijing as reference. The complete-subset base and indication-adjusted models use the same patient-years. These comparisons do not eliminate unmeasured differences in case mix or establish a causal explanation for retention.

**Supplementary Table S14. Survival diagnostics, horizon-specific cumulative return estimates, and multiplicity checks.**

A. Proportional-hazards checks and time-specific origin associations. All Cox models stratify baseline hazards by first-visit year and adjust for age and sex. This avoids estimating coefficients for sparse or zero-event calendar years. Schoenfeld tests use rank-transformed time and Efron ties for diagnostic computation; reported HRs use Breslow ties. Origin PH P values are BH-adjusted across model terms within each cohort. Later models condition on remaining event-free at 180 days. Not estimable denotes fewer than five later events in at least one origin group; these unstable estimates are not reported. The 180-day split is an exploratory sensitivity analysis, not a clinical threshold.

| **Cohort** | **PH P, nearby / distant** | **Time window** | **Nearby HR (95% CI)** | **Distant HR (95% CI)** |
| --- | --- | --- | --- | --- |
| overall | 0.196 / 0.001 | 0–180 days | 0.98 (0.88–1.10) | 0.94 (0.87–1.02) |
|  |  | >180 days | 0.87 (0.76–0.99) | 0.68 (0.62–0.76) |
| Neuromodulator-type | 0.599 / 0.599 | 0–180 days | 0.76 (0.58–0.98) | 1.04 (0.89–1.23) |
|  |  | >180 days | 0.81 (0.65–1.02) | 0.72 (0.62–0.85) |
| Volume/contour-type | 0.847 / 0.847 | 0–180 days | 0.85 (0.64–1.12) | 0.86 (0.71–1.06) |
|  |  | >180 days | 1.05 (0.59–1.87) | 1.10 (0.74–1.62) |
| Skin-quality-type | 0.740 / 0.740 | 0–180 days | 1.01 (0.81–1.26) | 0.79 (0.65–0.96) |
|  |  | >180 days | 0.99 (0.79–1.25) | 0.72 (0.60–0.87) |
| Post-procedure review | 0.920 / 0.840 | 0–180 days | 1.55 (1.15–2.09) | 1.44 (1.12–1.84) |
|  |  | >180 days | Not estimable | Not estimable |

B. Multiplicity sensitivity. Benjamini–Hochberg adjustment is applied separately within each indicated family of subgroup-origin comparisons. These adjusted P values do not convert exploratory analyses into confirmatory findings. The three principal interaction BH-adjusted P values are: survival <0.001, cadence = 0.012, and retention = 0.061.

| **Family** | **Comparison** | **Nominal P** | **BH-adjusted P** |
| --- | --- | --- | --- |
| Table 6 | Neuromodulator-type; Nearby nonlocal | 0.007 | 0.018 |
| Table 6 | Neuromodulator-type; Distant nonlocal | 0.011 | 0.018 |
| Table 6 | Volume/contour-type; Nearby nonlocal | 0.319 | 0.365 |
| Table 6 | Volume/contour-type; Distant nonlocal | 0.269 | 0.359 |
| Table 6 | Skin-quality-type; Nearby nonlocal | 0.987 | 0.987 |
| Table 6 | Skin-quality-type; Distant nonlocal | <0.001 | <0.001 |
| Table 6 | Post-procedure review; Nearby nonlocal | 0.010 | 0.018 |
| Table 6 | Post-procedure review; Distant nonlocal | 0.009 | 0.018 |
| S12 | Neuromodulator-type; Nearby nonlocal | 0.025 | 0.050 |
| S12 | Neuromodulator-type; Distant nonlocal | 0.002 | 0.007 |
| S12 | Volume/contour-type; Nearby nonlocal | 0.883 | 0.883 |
| S12 | Volume/contour-type; Distant nonlocal | 0.006 | 0.017 |
| S12 | Skin-quality-type; Nearby nonlocal | 0.118 | 0.149 |
| S12 | Skin-quality-type; Distant nonlocal | 0.043 | 0.069 |
| S12 | Post-procedure review; Nearby nonlocal | 0.001 | 0.007 |
| S12 | Post-procedure review; Distant nonlocal | 0.130 | 0.149 |
| S13 | 1 visit; Nearby nonlocal | 0.053 | 0.053 |
| S13 | 1 visit; Distant nonlocal | <0.001 | <0.001 |
| S13 | 2 visits; Nearby nonlocal | <0.001 | 0.001 |
| S13 | 2 visits; Distant nonlocal | <0.001 | <0.001 |
| S13 | >=3 visits; Nearby nonlocal | 0.009 | 0.014 |
| S13 | >=3 visits; Distant nonlocal | 0.017 | 0.020 |

C. Kaplan–Meier cumulative return probabilities with Greenwood log-log 95% confidence intervals. NE denotes a horizon beyond the observed follow-up support in that group; probabilities are not extrapolated. Other late probabilities based on sparse risk sets should also be interpreted cautiously. Counts at risk are shown in Figures 5 and 6.

| **Cohort** | **Origin** | **365-day return, % (95% CI)** | **1,095-day return, % (95% CI)** |
| --- | --- | --- | --- |
| overall | Beijing | 27.8 (27.1–28.5) | 34.7 (34.0–35.5) |
|  | Nearby nonlocal | 26.4 (24.4–28.5) | 32.9 (30.6–35.2) |
|  | Distant nonlocal | 24.2 (22.9–25.6) | 30.0 (28.5–31.5) |
| Neuromodulator-type | Beijing | 33.6 (32.2–35.1) | 43.9 (42.2–45.7) |
|  | Nearby nonlocal | 28.5 (24.3–33.1) | 37.0 (31.9–42.6) |
|  | Distant nonlocal | 30.0 (27.2–33.1) | 39.0 (35.6–42.6) |
| Volume/contour-type | Beijing | 30.9 (28.7–33.3) | 46.3 (31.5–64.1) |
|  | Nearby nonlocal | 25.5 (20.1–32.1) | NE |
|  | Distant nonlocal | 28.2 (24.2–32.7) | 55.2 (25.5–88.8) |
| Skin-quality-type | Beijing | 28.6 (27.3–30.1) | 37.6 (36.1–39.1) |
|  | Nearby nonlocal | 30.1 (25.9–34.7) | 39.8 (35.1–44.9) |
|  | Distant nonlocal | 24.2 (21.4–27.3) | 32.4 (29.1–35.8) |
| Post-procedure review | Beijing | 37.2 (32.6–42.2) | NE |
|  | Nearby nonlocal | 49.7 (41.1–59.1) | NE |
|  | Distant nonlocal | 47.3 (41.1–54.0) | NE |

**Supplementary analysis scripts**

Three Python scripts are provided as Supplementary Appendices S2–S4. Supplementary Appendix S2 (Supplementary_Appendix_S2_derivation.py) processes the existing nine-field encounter export. Supplementary Appendix S3 (Supplementary_Appendix_S3_analysis.py) constructs patient-year and survival measures and generates aggregate analyses and figures. Supplementary Appendix S4 (Supplementary_Appendix_S4_district_figure.py) generates the district-level patient-origin figure. The scripts do not include patient-level data or the hospital database query.

**FIGURES**

**Supplementary Figure S1**. Distribution of patient origins by province. Heatmap showing the number of unique patients by province in three periods (2015-2019, 2020-2022, and 2023-2024). Cell values indicate patient counts. Color intensity represents patient volume on a logarithmic scale. Provinces are grouped and labeled according to the six-region classification.

**Supplementary Figure S2**. Unique patients by Beijing district and period. A district-level heatmap summarizes 2015–2019, 2020–2022, and 2023–2024. Counts are descriptive and not population-standardized; a patient may appear in more than one period. Unresolved districts are shown separately. The institution’s precise location is not displayed.

**Supplementary Figure S3**. Annual visit-count distribution by patient origin, violin plots (2022-2024). Violin plots showing the distribution of annual visit counts per patient-year on a logarithmic y-axis, for (A) the three-level classification and (B) the six-region classification. The width of each violin reflects the density of observations and the white marker indicates the median.

**Supplementary Figure S4**. Exploratory billing-based cost per visit by patient origin (2022-2024). Box plots showing the distribution of cost per visit (RMB) per patient-year on a logarithmic y-axis, for (A) the three-level classification and (B) the six-region classification. Boxes indicate the median and interquartile range (IQR), whiskers represent 1.5 x IQR, and points denote outliers. Cost per visit was calculated as annual total cost divided by annual visit count; patient-years with any missing encounter charge or zero recorded total cost were excluded.
